# TEGylated Double-Walled Carbon Nanotubes as Platforms to Engineer Neuronal Networks

**DOI:** 10.1021/acsami.2c16808

**Published:** 2022-10-21

**Authors:** Myriam Barrejón, Francesca Zummo, Anastasiia Mikhalchan, Juan J. Vilatela, Mario Fontanini, Denis Scaini, Laura Ballerini, Maurizio Prato

**Affiliations:** † Department of Chemical and Pharmaceutical Sciences, INSTM, UdR Trieste, University of Trieste, Via L. Giorgieri 1, Trieste34127, Italy; ‡ Neural Repair and Biomaterials Laboratory, Hospital Nacional de Parapléjicos (SESCAM), Finca la Peraleda s/n, Toledo45071, Spain; § International School for Advanced Studies (SISSA/ISAS), Trieste34136, Italy; ∥ IMDEA Materials, Eric Kandel 2, Getafe, Madrid28906, Spain; ⊥ Basque Foundation for Science, Ikerbasque, Bilbao48013, Spain; # University of Basque Country, Faculty of Pharmacy, Paseo de la Universidad 7, Vitoria-Gasteiz01006, Spain; g Center for Cooperative Research in Biomaterials (CIC biomaGUNE), Basque Research and Technology Alliance (BRTA), Paseo de Miramon 194, Donostia San Sebastián20014, Spain

**Keywords:** double-walled carbon nanotubes, cross-linking, polymer-free 3D scaffolds, electronic properties, mechanical properties, neuronal growth, neuronal activity

## Abstract

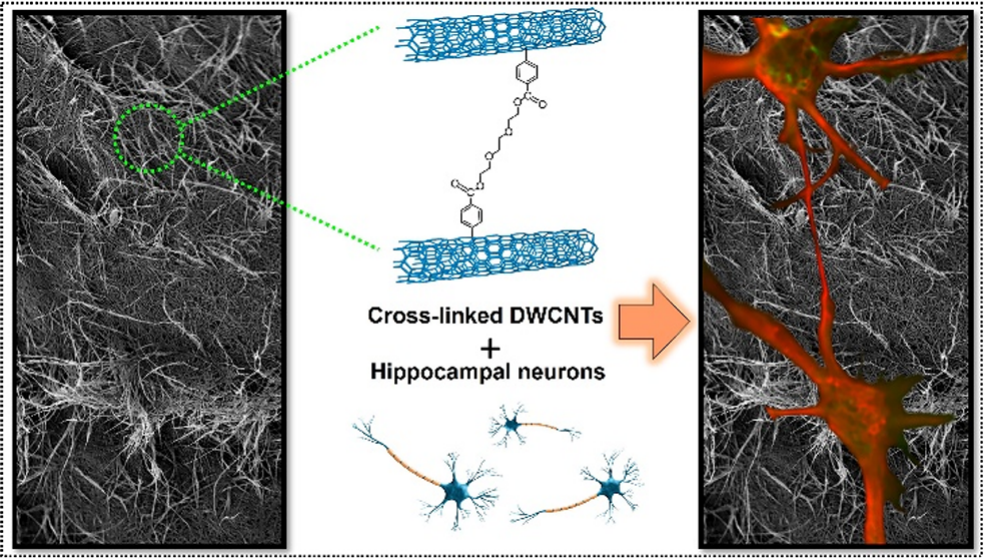

In the past two decades, important results have been obtained on the application of carbon nanotubes (CNTs) as components of smart interfaces promoting neuronal growth and differentiation. Different forms of CNTs have been employed as scaffolds, including raw CNTs and functionalized CNTs, characterized by a different number of walls, mainly single-walled CNTs (SWCNTs) or multiwalled CNTs (MWCNTs). However, double-walled carbon nanotubes (DWCNTs), which present interesting electronic and transport properties, have barely been studied in the field. Apart from the electrical conductivity, the morphology, shape, porosity, and corresponding mechanical properties of the scaffold material are important parameters when dealing with neuronal cells. Thus, the presence of open porous and interconnected networks is essential for cell growth and differentiation. Here, we present an easy methodology to prepare porous self-standing and electrically conductive DWCNT-based scaffolds and study the growth of neuro/glial networks and their synaptic activity. A cross-linking approach with triethylene glycol (TEG) derivatives is applied to improve the tensile performance of the scaffolds while neuronal growth and differentiation are promoted. By testing different DWCNT-based constructs, we confirm that the manufactured structures guarantee a biocompatible scaffold, while favoring the design of artificial networks with high complexity.

## 1 Introduction

A key goal of neuroscience is to combine tissue engineering with electrical interfacing to recover or rehabilitate lost central nervous system (CNS) functions, overcoming the effects of CNS lesions and diseases. In recent years, nanotechnology has played a leading role in the advancement of neuroscience, and the development of stimuli-responsive scaffold materials with a tunable design that mimics the extracellular matrix of the native tissue has been a primary objective.^1−3^ Efforts are being made to produce promising neural interfaces able to optimize efficiency and minimize side effects after implantation.^4−6^ Choosing the appropriate scaffold material is a critical issue in achieving better performance. A promising neural interface requires the combination of a variety of properties that are difficult to attain with conventional materials: reduced dimensions with a large surface area, appropriate charge delivering, optimal mechanical and electrochemical properties, and long-term biocompatibility.^6−8^ Metal electrodes have been used as neural interfaces since the early twentieth century; however, metals lack tissue-like features, such as porosity or elastic properties, preventing their translation into in vivo systems.^6,9^ It is well-known that CNTs possess high mechanical strength, flexibility, and electrical conductivity and there has been a strong encouragement for their application in the field of neuroscience. Furthermore, due to their extremely small size and high effective area, CNTs allow the preparation of small and flexible scaffolds that may integrate better within the neural tissue, improving the recording/stimulation of neural activity. Thus, during the past decades, CNTs have been widely explored to modulate neuronal behavior at either the structural or functional level.^10−15^ The properties of CNTs could be further tailored by appropriate chemical modifications of their wall to develop scaffold materials with the ideal configuration for recording and stimulation.^16,17^ In this sense, when modified with biologically active compounds or functionalized in order to alter their charge, CNTs were demonstrated to affect neurite outgrowth and branching.^18^

Among the different types of existing CNTs, double-walled carbon nanotubes (DWCNTs) form a special class of nanotubes that consist of two nanotubes, one nested within the other, where the interlayer distance is similar to that of graphene layers in turbostratic graphite.^19,20^ The most important feature of DWCNTs is the possibility of selectively functionalizing the outer nanotube without altering the mechanical and electrical properties of the inner nanotube.^21,22^ This could be a critical aspect for their applications in nanoelectronics or electrically conductive neural probes or implantable devices,^23^ as the electrical properties of the inner tubes can be retained even after heavy functionalization of the outer wall via covalent approaches. As such, in recent years, there has been a growing interest in the application of DWCNTs in different research areas, such as transparent conducting thin films,^24,25^ field-effect transistors,^26,27^ energy storage devices,^28^ solar cells,^29,30^ and sensors.^31^ However, though several papers have reported the use of single-walled carbon nanotubes (SWCNTs) and multiwalled carbon nanotubes (MWCNTs) in neural interfaces,^4,6,10,32−34^ the application of DWCNTs in neuroscience remains fairly unexploited despite their advantages over their single-walled and multiwalled counterparts. For example, in addition to the aforementioned superior electrical properties, DWCNTs exhibit an increase in thermal and chemical stability over SWCNTs.^20^ As compared to MWCNTs, DWCNTs have smaller dimensions, demonstrate lower interwall resistance, and have better transparency-conductance performance.^20,35^ These properties have proved quite enticing to the development of electrically conductive scaffold materials for neural interfaces.^36^

Coupled with the inherent mechanical characteristics of constituent CNTs, their 3D ensembles could enable robust scaffolds with balanced mechanical strength and electrical conductivity and a sufficient degree of flexibility to withstand mechanical handling at insertion surgery and—equally important—strains at postural movements experienced afterward. Among the four main characteristic mechanisms of primary injury of the spinal cord at traumatic events the impact plus persistent compression (i.e., compressive damage) are the most common. However, in cases of missile injuries or sharp bone fragment dislocations, the complete laceration/transection of the spinal cord could happen (i.e., tensile fracture).^37^ In addition, secondary cellular changes followed the injury induce swelling, which itself can lead to further compression and, therefore, worsen the injury.^38^ Despite their importance, the mechanical properties are poorly characterized for nanocarbon-based scaffolds, with rare reports in the literature on compressive^39,40^ rather than tensile performance. However, even though it is not a predominant mechanism involved in spinal cord injury, tensile testing is the most common biomechanical test on the spinal cord.^23^ During normal postural movements, the spinal cord could experience significant deformations, with the tensile strains able to reach enormous levels of 10–20%;^41,42^ therefore, tensile stress–strain behavior and structural elasticity of the scaffolds are especially relevant for neural restorations. Tensile properties also give a measure of the overall ductility and toughness. In addition, the mechanical properties of the cell environment have a huge impact on cell growth and behavior. In general, the pores of the 3D CNT ensemble need to be large enough to allow cell migration, but not too large to compromise the mechanical properties of the scaffold, matching the viscoelastic nature of neural tissue.^43^ Therefore, mechanical properties, both in tension and compression, need to be carefully considered.

Recent advances in biointerface technologies based on CNTs require engineering their properties via chemical manipulations. In this regard, we have recently shown that cross-linking of SWCNTs allows for obtaining growth substrates consisting of conductive CNT films characterized by irregular porosity.^16^ In this work, we have extended the cross-linking approach to DWCNTs, including an additional step to develop polymer-free 3D conductive scaffolds and probe neural circuit development in a three-dimensional fashion. For the sake of comparison, pristine DWCNT-based 3D scaffolds and two control materials based on non-cross-linked DWCNT 3D scaffolds with similar functionalization approaches have been included in the study. We have evaluated DWCNT-based scaffolds’ mechanical properties in compression and tension and the ability to sustain the growth of neuro/glial networks tightly wrapped on the curved surfaces of the foams. Primary mammalian neurons and glial cells have been isolated from the hippocampus of neonatal rats and seeded on the various substrates. By live calcium imaging and immunofluorescence confocal microscopy, used to monitor synaptic activity and network formation, we have documented the functional development and clustering of small-scale, highly connected networks only when interfacing hippocampal cells to cross-linked DWCNT 3D scaffolds.

## 2 Experimental Section

### 2.1 Materials

All the chemicals and solvents were purchased from Sigma-Aldrich and used without any further purification. Double-walled carbon nanotubes were purchased from Xinanno Materials (catalog number: XNM-UP-11050, purity >98%), average length >10 μm, outer and inner diameters 1–1.81 and 0.88 nm, respectively.

### 2.2 Characterization Methods

Raman spectra were obtained on a Renishaw inVia Raman microscope at room temperature with an exciting laser source (λ = 785 cm^–1^). Measurements were taken with 10 s of exposure time, and the laser spot was focused on the sample surface using a long working distance of 50× objective. Raman maps were recorded for pristine and X-DWCNTs to create statistical histograms of the *I*_D_/*I*_G_ ratio. Thermogravimetric analyses were performed with a TGA Q500 (TA Instruments) at 10 °C/min under N_2_. Scanning electron microscopy (SEM) images were acquired by collecting secondary electrons on a commercial SEM (Gemini SUPRA 40, Carl Zeiss NTS GmbH, Oberkochen, Germany). For the analysis, the different materials were placed on conductive double-sided carbon tape (Ted Pella, Inc., USA) and imaged at 5 keV. A piece of 3D material was sectioned using a scalpel and mounted on the SEM stub using double-sided carbon tape. Sheet resistance measurements of the thin films were carried out by four-point probe technique using a Jandel four-point probe analyzer (RM-3000). For this purpose, the required films were prepared through filtration of 1 mg of material on a PTFE filter (Millipore 0.45 μm pores), and the membrane thickness was measured with a micrometer (HighAccuracy Digimatic micrometer 293–100, Mitutoyo). The conductivity values of the thin films were calculated from the measured sheet resistances according to the formula σ = 1/(*R*_s_*t*), where σ was the electrical conductivity, *R*_s_ the sheet resistance, and *t* the thickness of the sheets. Conductivity values of the 3D scaffolds were measured directly after their manufacturing process using the same formula, while the thickness was determined from the SEM cross-section images.

#### Mechanical Characterization of DWCNT-Based Scaffolds

Mechanical tests were performed using the Dynamic Mechanical Analyzer machine (DMA 850, TA Instruments) equipped with the 18 Newton load cell and clamps designed for uniaxial tests in tension and compression. Tensile tests were performed on the samples with an initial gauge length of 10 mm in the force-controlled mode at 1 N/min ramp, which corresponds to the quasistatic regime. The CNT scaffolds were freestanding and robust to survive cutting by a sharp blade into rectangular strips with a width of 3 to 4 mm, while retaining the original shape without visible signs of premature structural damage (Figure S9). To avoid stress concentration at the grips and to prevent sample slippage, both ends of each specimen were fixed in the cardboard frame with fast-cure epoxy adhesive. The load–displacement curves were recorded, and the corresponding absolute and specific values of tensile strength, elastic modulus, and elongation to break were calculated afterward. The cross-sectional areas were determined by the SEM analysis, and the mass of each specimen was measured using high precision microbalance to calculate the apparent volumetric density.

The compression tests were performed under the controlled force regime applying the lower force ramp of 0.3 N/min. Rectangular samples with the dimensions of 3 × 5 mm were cut from the CNT scaffolds (one representative sample of each type of scaffolds), placed between the loading plates of the machine (Figure S9), and tested under ambient conditions. The rectangular shape and flat surface of the samples ensured the compressive stress has been evenly distributed over the surface while in contact with the plates. The displacement was recorded automatically by the software in relationship to the scaffold′s initial thickness. The scaffolds were compressed until the stabilization of the displacement in time (reaching the plateau, Figure S10) as an indication of the maximum possible compressibility and entire collapse of the scaffold′s structure. The compressive stress and modulus were calculated with the specimens’ initial dimensions in absolute (MPa) values without normalization to the apparent volumetric density because it was not constant and increased under compression. The modulus was calculated for the linear elastic section of the stress–strain curve in the initial stage of compressive deformation (2–4% strain interval).

### 2.3 Synthetic Methods

#### Synthesis of Ethanol, 2,2′-[1,2-Ethanediylbis(oxy)]bis-, bis(4-aminobenzoate) (Diamine 1)

A two-necked round-bottom flask was charged with 4-aminobenzoic acid (7.29 mmol) and 50 mL of Dimethylformamide (DMF). The mixture was stirred to obtain a homogeneous solution and then triethylene glycol di(*p*-toluenesulfonate) (3.65 mmol) and anhydrous potassium carbonate (21.87 mmol) were added to the solution. The mixture was stirred under nitrogen at 50 °C for 6 h, cooled to room temperature, and concentrated. The residue was dissolved in CH_2_Cl_2_ and washed with water and brine, the organic phase was dried over Na_2_SO_4_ and filtered, and the solvent was evaporated under reduced pressure. Column chromatography (silica gel, CHCl_3_/MeOH = 9:1) yielded the desired pure product as a white solid (1.1 g, 78%). ^1^H NMR (400 MHz, CDCl_3_, δ/ppm): 7.86 (dd, 4H), 6.62 (dd, 4H), 4.40 (t, 4H, *J* = 7.4 Hz), 4.05 (br s, 4H, NH_2_), 3.81 (t, 4H, *J* = 7.4 Hz), 3.71 (s, 4H).

#### Synthesis of 2-(2-(2-Methoxyethoxy)ethoxy)ethyl 4-methylbenzenesulfonate (Tos-TEG)

Triethylene glycol monomethyl ether (10 g, 60.9 mmol) in 100 mL of tetrahydrofuran (THF) was added dropwise to a solution of NaOH (3.65 g, 91.3 mmol) in 40 mL of THF/water (1/1) at 0 °C. The mixture was stirred for 15 min before the dropwise addition of *p*-toluenesulfonyl chloride (12.7 g, 67 mmol) in 20 mL of water. After being stirred for 3 h at room temperature the mixture was poured onto ice and extracted with 150 mL of CH_2_Cl_2_ (3 × 50 mL). The resulting organic layer was subsequently dried over Na_2_SO_4_, filtered, and the solvent was evaporated under reduced pressure yielding a yellow oil (15 g, 83%) that was used without further purification. ^1^H NMR (400 MHz, CDCl_3_, δ/ppm): 7.78 (d, 2H, *J* = 8.2 Hz), 7.32 (d, 2H, *J* = 8.0 Hz), 4.12 (t, 2H, *J* = 4.8 Hz), 3.71–3.52 (m, 10H), 3.33 (s, 3H), 2.41 (s, 3H).

#### Synthesis of 2-(2-(2-Methoxyethoxy)ethoxy)ethyl 4-aminobenzoate (Amino-TEG 2)

2-(2-(2-Methoxyethoxy)ethoxy)ethyl 4-methylbenzenesulfonate (5 g, 15.7 mmol) was dissolved in DMF (50 mL) and the solution was purged with argon for 10 min. K_2_CO_3_ (6.5 g, 47 mmol) and 4-aminobenzoic acid (2.15 g, 15.7 mmol) were added and the mixture was stirred for 24 h at 50 °C, cooled to room temperature and concentrated. The residue was dissolved in CH_2_Cl_2_ and washed with water, and the organic phase was dried over Na_2_SO_4._ The final product (Amino-TEG **2**) was obtained as a yellow oil (4.1 g, 92%) and used without further purification. ^1^H NMR (400 MHz, CDCl_3_, δ/ppm): 7.64 (d, *J* = 8.0 Hz, 2H), 6.47 (d, *J* = 8.0 Hz, 2H), 4.35 (br s, 2H, NH_2_), 4.24 (t, *J* = 4.0 Hz, 2H), 3.60 (t, *J* = 4.0 Hz, 2H), 3.55 (t, *J* = 4.0 Hz, 2H), 3.40–3.52 (m, 4H) 3.35 (t, *J* = 4.0 Hz, 2H), 3.20 (s, 3H).

#### Synthesis of bis(2-(2-Methoxyethoxy)ethyl) 5-aminoisophthalate (Amino-TEG 3)

2-(2-(2-Methoxyethoxy)ethoxy)ethyl 4-methylbenzenesulfonate (5 g, 15.7 mmol) was dissolved in DMF (50 mL) and the solution was purged with argon for 10 min. K_2_CO_3_ (6.5 g, 47 mmol) and 5-Aminoisophthalic acid (2.8 g, 15.7 mmol) were added, and the mixture was stirred for 48 h at 50 °C, cooled to room temperature and concentrated. The residue was dissolved in CH_2_Cl_2_ and washed with water, and the organic phase was dried over Na_2_SO_4._ The resulting product was purified by column chromatography (silica gel, CHCl_3_/MeOH = 9:1) yielding Amino-TEG **3** as a yellow oil (4.8 g, 80%).^1^H NMR (400 MHz, CDCl_3_, δ/ppm): 8.07 (t, *J* = 1.4 Hz, 1 H), 7.48 (d, *J* = 1.7 Hz, 2 H), 4.45 (t, *J* = 4.9 Hz, 4 H), 4.05 (br s, 2 H, NH_2_), 3.78 (t, *J* = 4.8 Hz, 4 H), 3.72–3.60 (m, 12 H), 3.56–3.50 (m, 4 H), 3.30 (s, 6 H).

#### General Procedure for the Synthesis of Functionalized DWCNTs (X-DWCNT, Y-DWCNT, and Z-DWCNT)

One-hundred milligrams of pristine DWCNTs were stirred for 24 h in 150 mL of DMF and sonicated for 3 h, keeping the temperature below 35 °C during the sonication process. 1.2 mmol of the corresponding amine and 5 mmol of isopentyl nitrite were added to the dispersion and the reaction mixture was stirred for 48 h at 80 °C. The mixture was filtered through a PTFE membrane with an average pore size of 0.45 μm, and the black precipitate was washed several times with DMF, methanol, and diethyl ether.

#### Synthesis of Cross-linked DWCNTs (X-DWCNTs)

According to the general procedure, DWCNTs (100 mg), diamine **1** (466 mg), and isopentyl nitrite (0.7 mL), gave the desired product as a black solid (102 mg).

#### Synthesis of Control Material 1 (Y-DWCNTs)

According to the general procedure, DWCNTs (100 mg), amino-TEG **2** (340 mg), and isopentyl nitrite (0.7 mL), gave the desired product as a black solid (108 mg).

#### Synthesis of Control Material 2 (Z-DWCNTs)

According to the general procedure, DWCNTs (100 mg), amino-TEG **3** (568 mg), and isopentyl nitrite (0.7 mL), gave the desired product as a black solid (107 mg).

### 2.4 Preparation of 3D Porous Scaffolds

The manufacturing of the 3D porous scaffolds was performed through a multistage procedure. 50 mg of DWCNTs (pristine, X-DWCNT, Y-DWCNT, or Z-DWCNT) were dispersed in THF (5 mL) using an ultrasonic batch. Then, 250 mg of food-grade sodium chloride (NaCl) was grounded by using stainless steel mesh sieve (mesh size 100 μm) (Fisher Scientific Inc.), and 5 mg of the as-ground NaCl was added to the DWCNT dispersion and sonicated. The mixture was vacuum filtered through a PTFE membrane with an average pore size of 0.45 μm using a typical glass microfiltration setup. The resulting materials were peeled-off by tweezers and immersed in water for 48 h to remove the NaCl crystals. During this step, to facilitate the immersion of the 3D scaffolds, these were sunk into the water with the help of tweezers. They were then dried in a vacuum oven at 40 °C for 24 h to yield the desired 3D porous DWCNT-based scaffolds.

### 2.5 Primary Hippocampal Cultures

All experiments were performed in accordance with the EU guidelines (2010/63/UE) and Italian law (Decree 26/14) and were approved by the local authority veterinary service and by our institution (SISSA) animal wellbeing committee (OBPA). All efforts were made to minimize animal suffering and to reduce the number of animals used. Animal use was approved by the Italian Ministry of Health (22DABNQYA), in agreement with the EU Recommendation 2007/526/CE.

Dissociated hippocampal cells were obtained from postnatal day P2–P4 old rats, as previously described.^44^ Briefly, hippocampi were isolated from rat pups’ brains, digested in trypsin (6000 U/mL, Sigma-Aldrich) and deoxyribonuclease (1560 U/mL, Sigma-Aldrich). After chemical and mechanical digestion, the solution containing cells was centrifuged at 800 rpm for 5 min and the pellet was resuspended in a culture medium. DWCNT samples were treated with air plasma-cleaner and sterilized with UV, 1 h before plating cells. Cells were seeded on pristine and functionalized DWCNTs scaffolds; the samples were incubated (5% CO_2_ at 37 °C) for 1 h in order to stabilize cell adhesion. The plating cell density was 500 cells/mm^2^. The cells were then maintained in a humidified incubator at 37 °C in the presence of 5% CO_2_ and grown in Neurobasal medium (Thermofisher) with B-27 supplement (2%, ThermoFisher), Glutamax (10 mM, Thermofisher), and Gentamycin (500 nM, Thermofisher). All experiments were performed at 8–10 days *in vitro* (DIV).

### 2.6 Immunofluorescence and Confocal Microscopy

Cultures were fixed with 4% formaldehyde (PFA, prepared from fresh paraformaldehyde; Sigma) in PBS (1×) for 20 min at room temperature (RT) and washed in PBS. Cultures were permeabilized and blocked in PBS, 5% FBS (Sigma) and 0.3% Triton X-100 (Sigma) at RT for 1 h. After rinsing, primary antibodies were added for 1 h at RT and, subsequently, secondary antibodies for 45 min. The primary antibodies used were: rabbit polyclonal anti-β-tubulin III (Sigma-Aldrich, 1:250 dilution), mouse monoclonal anti-GFAP (Sigma-Aldrich, 1:250 dilution). Secondary antibodies used were AlexaFluor 594 goat anti rabbit (Invitrogen, dilution 1:500), AlexaFluor 488 goat anti mouse (Invitrogen, dilution 1:500), DAPI (Invitrogen, dilution 1:200) to stain the nuclei. To label actin filaments, AlexaFluor 488 Phalloidin (Invitrogen, dilution 1:500) was used. Samples were mounted in Fluoromont-G (ThermoFisher). Images were acquired with an inverted confocal microscope (Nikon A1R) using 40× dry objective (NA 0.95), with z-stacks taken every 0.75 μm, and 60× oil objective (NA 1.4) with z-stacks taken every 200 nm.

### 2.7 Calcium Imaging

Hippocampal cultures were loaded with cell-permeable Ca^2+^ dye Oregon Green 488 BAPTA-1 AM (Molecular Probes). A 4 mM dye solution was prepared by adding 10 μL of DMSO (Sigma-Aldrich) to the 50 μg of stock solution, and cultures were incubated with a final dye concentration of 4 μM for 40 min at 37 °C, 5% CO_2_. The sample was then mounted in a fixed-stage upright microscope (Eclipse FN1, Nikon). Cultures were continuously perfused at 5 mL/min rate at RT with extracellular saline solution of the following composition (in mM): 150 NaCl, 4 KCl, 1 MgCl_2_, 2 CaCl_2_, 1 MgCl_2_, 10 HEPES, 10 glucose (all Sigma), pH 7.4. Ca^2+^ dye was excited at 488 nm with a mercury lamp; excitation light was separated from the light emitted from the sample using a 505 nm dichroic mirror and ND filter (1/32). Spontaneous calcium transients were recorded with a 20× water immersion objective (Fluor, 0.50 W NA, Nikon) using an EMCCD camera (iXon Ultra 897, Andor, Oxford Instruments) controlled by a computer through NIS-elements D (Nikon) software. Images were acquired every 150 ms at 10 MHz readout compensating the read noise with ×300 EM gain. Spontaneous activity was therefore recorded. In order to weaken synaptic inhibition^14,44^ 5 μM gabazine (Sigma-Aldrich; 15–20 min), diluted in the saline solution, was added after 10 min of recording. Finally, 1 μM tetrodotoxin (TTX, Latoxan; 15–20 min), which is a voltage-gated fast Na^+^ channel blocker, was added to confirm the neuronal nature of the recorded signals.^44^ Recorded images were analyzed offline by Fiji (selecting region of interest, ROI, around cell bodies) and Clampfit (*pClamp* software, 11.0.3 version; Axon Instruments). Intracellular Ca^2+^ transients were detected as signals that exceed at least five times the standard deviation of the noise and were expressed as fractional amplitude increase (Δ*F*/*F*_0_, where *F*_0_ is the baseline fluorescence level and Δ*F* is the rise over baseline). The inter events interval (IEI), the time between the onset of consecutive Ca^2+^ events, was then calculated. IEI values recorded under the same experimental conditions were pooled together and averaged for further comparison. The correlation between the Ca^2+^ events among all cells recorded from the same field was assessed by cross-correlation analysis, as previously described.^44^

All data are reported as mean ± SEM (*n* is the number of cells, if not otherwise indicated). Statistical analyses were performed by the software Prism 6 (GraphPad Software). Normality was evaluated by Shapiro–Wilk test. Student’s *t* test and Mann–Whitney were performed for parametric and nonparametric data, respectively. *P* < 0.05 was considered to indicate a statistically significant difference. For IEI, all data are plotted as frequency distribution, and a Kolmogorov–Smirnov test was performed.

## 3 Results and Discussion

### 3.1 Features and Chemical Modification of the Starting DWCNTs (Pristine DWCNTs)

**Figure 1:**
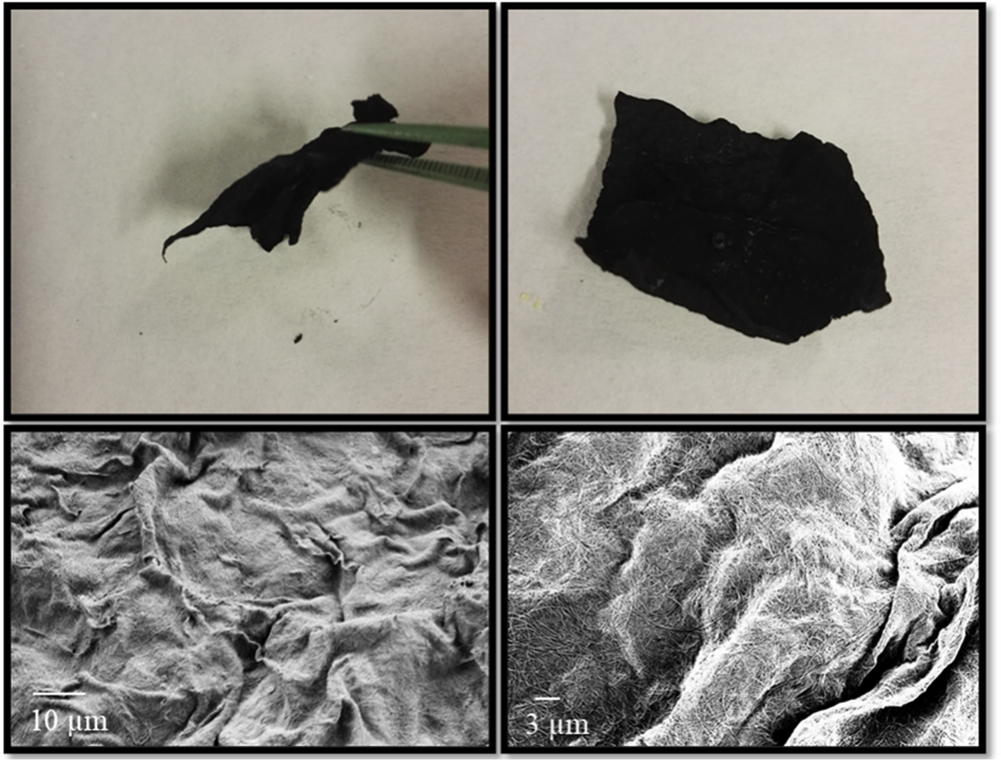
Top: Photographs of pristine DWCNT buckypaper; bottom: representative SEM images of pristine DWCNT buckypaper with low and high magnification from the top view.

The choice of the type of CNTs was based on their mechanical behavior and electroconductive properties since these are considered crucial factors for the development of scaffolds with long-term stability and optimal charge transfer capacity. The employed CNTs consisted of networks of long, ultrapure, and very entangled DWCNTs that resulted in a strong paper-like material with a rough surface and enough consistency to provide self-standing structures where no additives are needed. These features are easily observable in the scanning electron microscopy (SEM) images (Figure 1). A deeper insight into the mechanical properties of the DWCNTs is provided below (section 3.3). Studies on their electrical behavior were performed using a Jandel four-point probe analyzer (RM-3000), and the electrical conductivity (σ) was calculated from the measured sheet resistance (*R*_s_) and the thickness of DWCNT films prepared through the vacuum filtration method (*t*), according to the formula σ = 1/(*R*_s_*t*). The results revealed the presence of highly conductive DWCNTs, with an electrical conductivity ∼30 times higher than that of the SWCNTs employed in our former studies (σ = 3389 S/m for pristine SWCNTs versus 110525 S/m for pristine DWCNTs).^16^

**Scheme 1:**
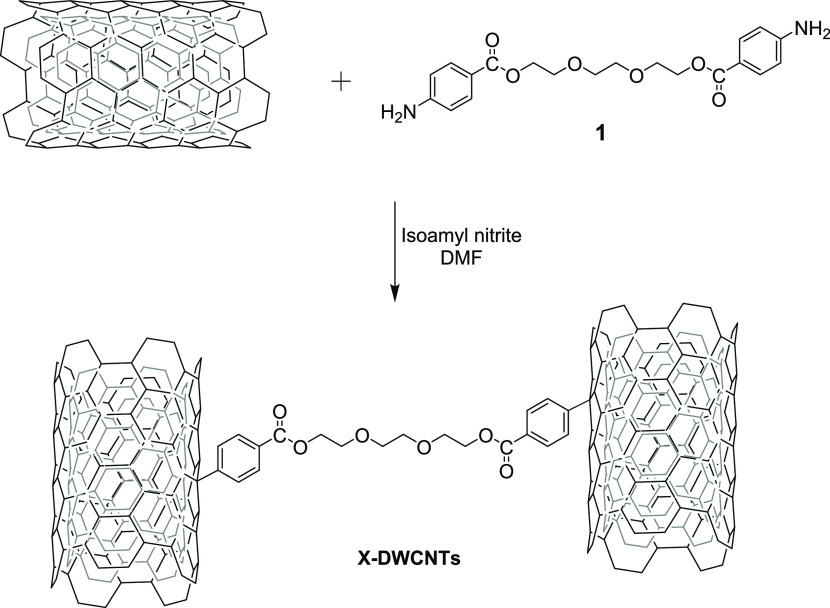
Synthetic Strategy for the Preparation of TEG-Cross-linked DWCNTs (X-DWCNTs)

These promising properties encouraged us to prepare new scaffolds based on DWCNTs for neural regeneration applications. Due to the entangled nature of the DWCNTs, easily noticeable in the high-magnification SEM images (Figure 1, bottom part right), their dispersion in organic solvents for further processing and preparation of scaffolds was a challenging task. In order to achieve more homogeneous dispersions, while at the same time the properties of the DWCNTs-based materials were tailored, we employed covalent chemical modification of the DWCNT sidewalls with triethylene glycol (TEG) derivatives. Furthermore, with the aim of engineering compact and porous structures with enhanced mechanical stability, the chemical modification of the DWCNTs was conducted through cross-linking approaches. For this purpose, aryl diazonium salt chemistry, which has been a powerful tool for grafting aryl groups to the surface of CNTs, was exploited for the chemical cross-linking procedure and a TEG derivative was employed as cross-linker (Scheme 1). Briefly, following a procedure described in the literature,^45^ the diamine **1** was prepared by a substitution reaction between the commercially available triethylene glycol di(*p*-toluenesulfonate) and *p*-aminobenzoic acid. Then, the resulting diamine was reacted with ultrapure DWCNTs through aryl diazonium salt chemistry^46^ to yield the desired cross-linked material (X-DWCNTs). The purification of the final networked material was performed by the common washing/filtration/sonication workup procedure (see the Experimental Section for further details).

In order to set a series of control conditions and reinforce the role of cross-linked materials as promising neuronal scaffolding platforms with enhanced stability, we prepared two control materials, hereinafter referred to as Y-DWCNTs and Z-DWCNTs (see Scheme S1).

Aryl diazonium salt chemistry^46^ was again employed for the preparation of aryl-substituted DWCNTs with a single TEG chain, in the case of Y-DWCNTs, or two TEG chains, in the case of Z-DWCNTs. These two derivatives enabled us to assess not only the impact of CNT cross-linking on 3D scaffolds’ mechanical properties, but also TEG contribution to neural cell behavior. Similarly to the case of diamine **1**, the synthesis of amino-TEG **2** and amino-TEG **3** was accomplished following a slightly modified procedure described in the literature^45^ (see the Experimental Section for further details). Briefly, the tosylate ester derivative (Tos-TEG) was prepared according to the method described by Hooper et al.^47^ from tosyl chloride and the glycol derivative in the presence of a strong base. Then, Tos-TEG was reacted with the corresponding amine (**2** or **3**) through a substitution reaction to yield the desired amino-TEG derivatives (Amino-TEG **2** and Amino-TEG **3**) (see Scheme S2).

### 3.2 Characterization of the Functionalized DWCNT-Based Materials and Fabrication of 3D Structures

**Figure 2:**
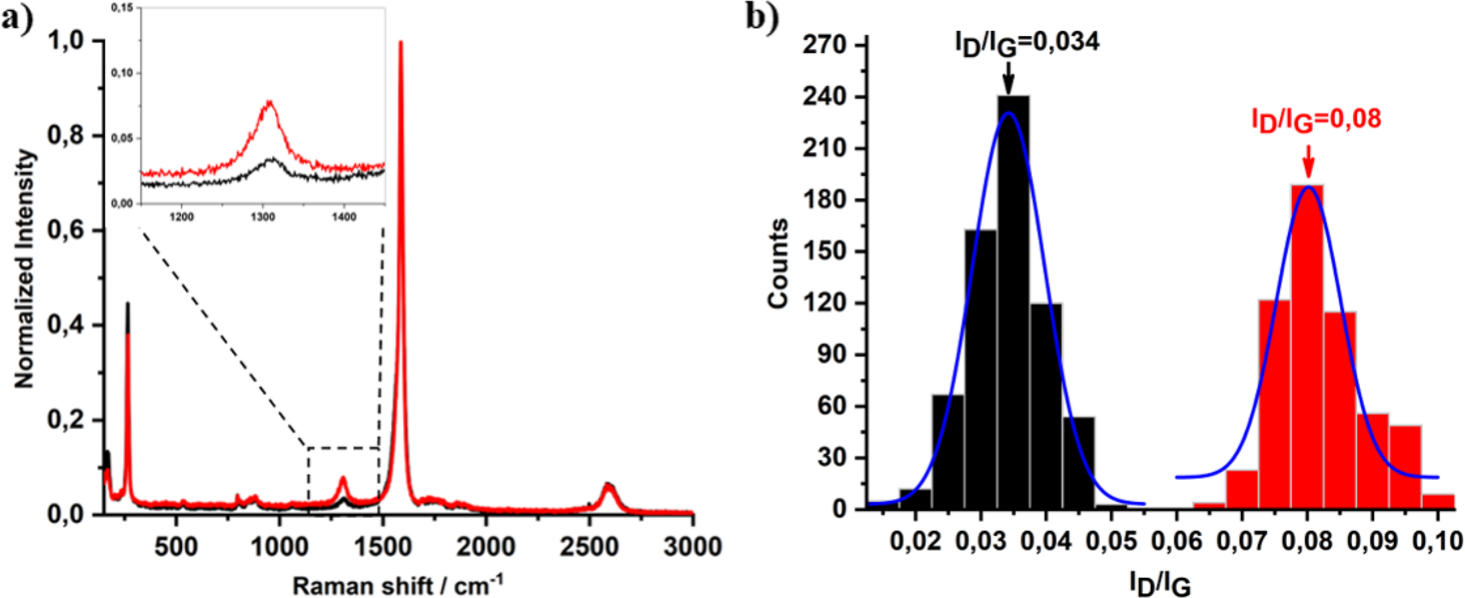
(a) Extended Raman spectrum and zoomed D band in the inset, and (b) statistical Raman *I*_D_/*I*_G_ histograms and corresponding Gauss distribution of the DWCNTs before (black) and after (red) the cross-linking process.

The successful functionalization of DWCNTs with TEG cross-linkers to yield X-DWCNTs was initially confirmed by Raman spectroscopy (Figure 2). Due to the high purity of the starting DWCNTs, the intensity of the D band was very low and its increase after the cross-linking process was not very noticeable (Figure 2a). To strengthen the evidence of D-band increase, statistical Raman spectroscopy was done. Raman maps with a step size of 2.5 μm were acquired for pristine and X-DWCNTs (Figure S1) and the corresponding histograms were generated (Figure 2b). The results developed an increase in the D band intensity from 0.034 for the pristine DWCNTs, to 0.08 for the cross-linked DWCNTs, confirming the higher content of sp^3^-bonded carbon atoms due to the covalent cross-linking process applied. Actual evidence of successful cross-linking was confirmed later during the development of mechanical studies.

A similar increase of the D band was observed in the extended Raman spectra of the control materials Y-DWCNTs (*I*_D_*/I*_G_ = 0.06) and Z-DWCNTs (*I*_D_*/I*_G_ = 0.07) (see Figure S2), confirming the successful covalent modification of the DWCNTs with TEG derivatives.

**Figure 3:**
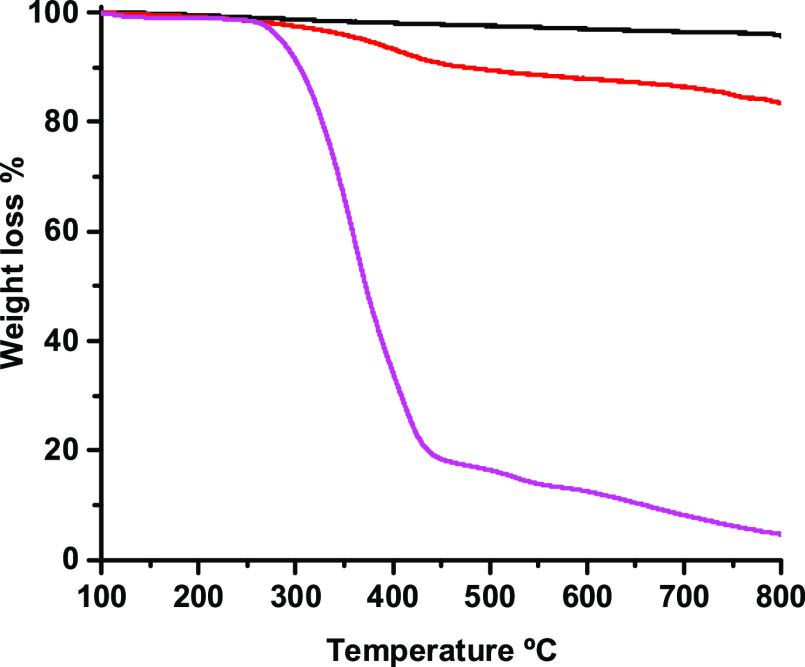
TGA curves of pristine DWCNTs (black) and X-DWCNTs (red), together with the thermal decomposition of diamine **1** recorded under inert conditions (magenta).

Further evidence of the successful functionalization of the DWCNTs via the TEG derivatives was achieved by thermogravimetric analysis (TGA) and consisted of heating ramps of 10 °C/min up to 800 °C, performed under nitrogen flow (Figure 3 and Figure S3). The weight loss measured for the thermal decomposition of the TEG derivatives at 550 °C was 12% for X-DWCNTs, 11.2% for Y-DWCNTs, and 10.4% for Z-DWCNTs compared to 3% for the starting DWCNTs. Furthermore, TGA analysis allowed us to estimate the functional group coverage (FGC) for the functionalized materials, which was calculated from the weight loss percentages of the attached TEG derivatives and DWCNTs, and the molecular weight of the TEG derivatives and atomic mass of carbon, according to the equation previously described in the literature:^16,48^
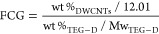
Using the above equation, the TGA curves developed the presence of one functional group every 260 carbon atoms for X-DWCNTs, one functional group every 203 carbon atoms for Y-DWCNT, and one functional group every 315 carbon atoms for Z-DWCNTs.

It is well-known that the electrical properties of CNTs are significantly affected by covalent functionalization.^49−51^ To assess how our functionalization approaches affected the electrical conductivity of the pristine DWCNTs, new conductivity studies were performed with the TEG-functionalized DWCNTs and compared to those of pristine DWCNTs. Our results highlight that the electrical conductivity decreased 1 order of magnitude after the functionalization processes (σ = 16230 S/m for X-DWCNTs, σ = 11345 S/m for Y-DWCNTs, and σ = 17282 S/m for Z-DWCNTs, versus 110525 S/m for pristine DWCNTs); however, despite the worsening of the electrical properties, TEG-functionalized DWCNTs showed significantly higher electroconductivity than that observed for the cross-linked SWCNTs employed as neural substrates in our former studies,^16^ which opened up a promising avenue for the development of electroactive cell scaffolds based on chemically modified DWCNTs.

**Scheme 2:**
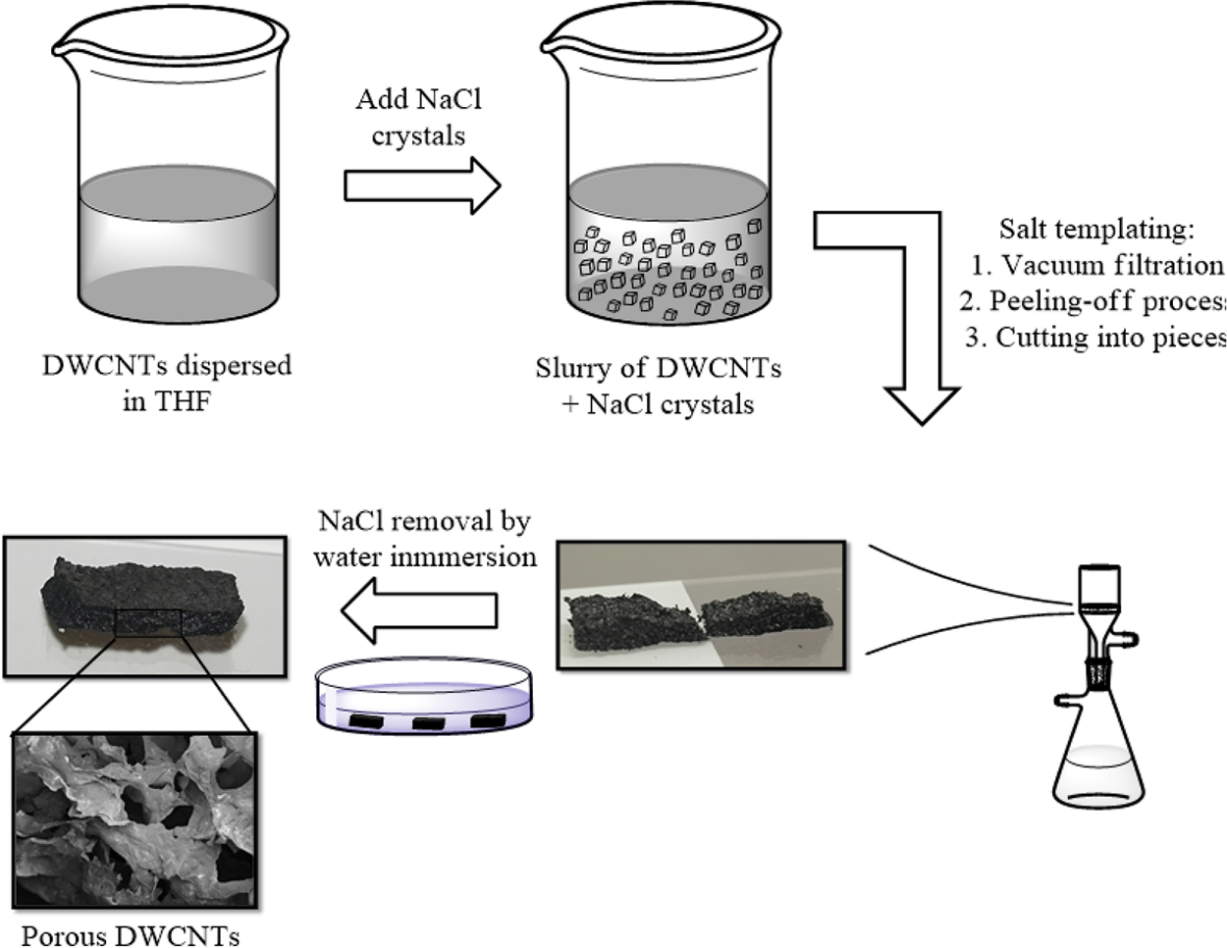
Schematic Showing the Preparation Process of the Porous DWCNT-Based Materials and SEM Image of the Resulting Porous Structure at the Bottom

As the ability of 3D porous materials to improve functional organization and synchronization of small neuronal assemblies has been previously demonstrated,^14,44,52^ we next developed an approach to manufacture 3D self-standing porous-conductive scaffolds based on our TEG-functionalized DWCNTs (X-DWCNTs, Y-DWCNTs, and Z-DWCNTs) and pristine DWCNTs. In this sense, we avoided the use of polymers in our 3D constructs, as the presence of polymers could have a negative effect on the intrinsic properties of CNTs and, moreover, the electrical conductivity of polymer composite materials is often very low.^53^ A summary of the fabrication process of our 3D structures is illustrated in Scheme 2. Our strategy consisted in creating microporous pure CNT foams by using sodium chloride (NaCl) crystals as templates, through a polymer-free method. In brief, DWCNTs were dispersed in tetrahydrofuran (THF) to form a slurry and sieved NaCl crystals were added to the dispersion, yielding a homogeneous mixture by sonication. The final polymer-free porous 3D materials were obtained through the well-known vacuum filtration method,^54^ using a typical glass microfiltration setup. Finally, the NaCl crystals were removed through a 48 h water immersion technique, yielding an interconnected 3D porous structure (see Experimental Section for further details).

### 3.3 Characterization of DWCNT-Based 3D Porous Materials

**Figure 4:**
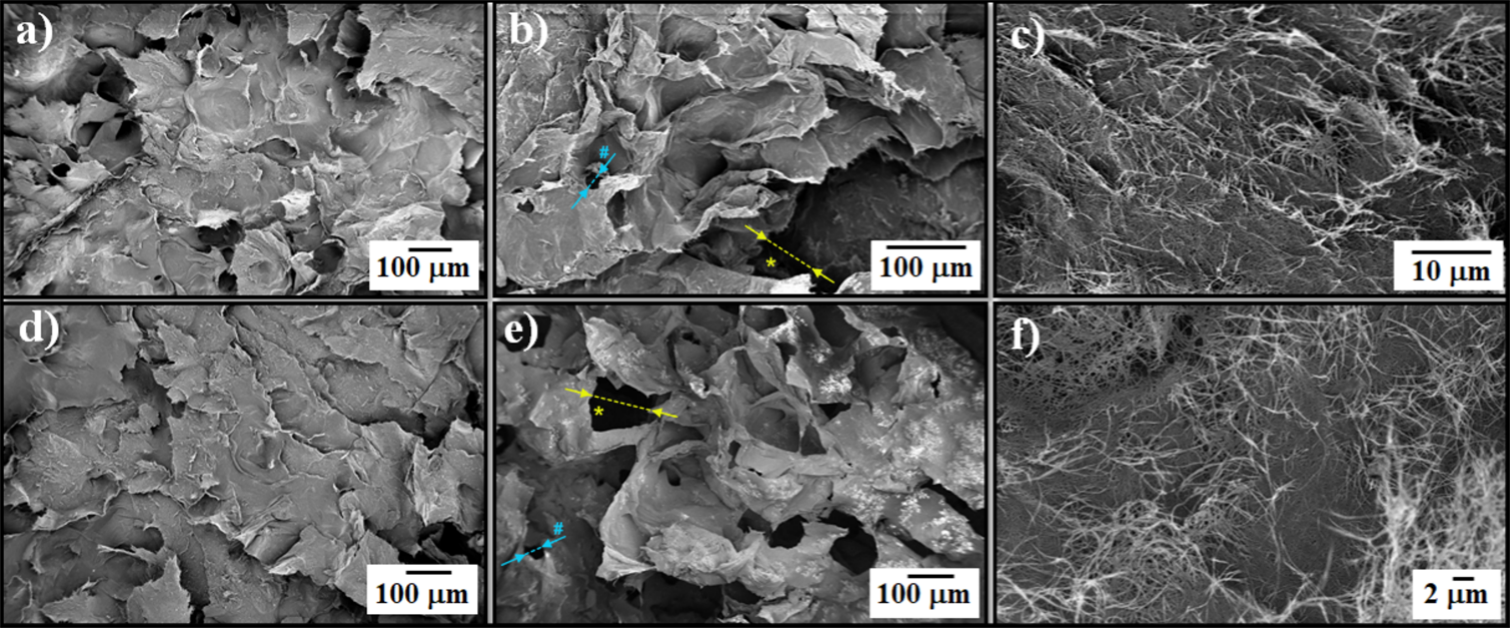
SEM images of manufactured porous scaffolds: (a) Top view of pristine DWCNTs; (b) sectional view of pristine DWCNTs; (c) sectional view of pristine DWCNTs with high magnification; (d) top view of X-DWCNTs; (e) sectional view of X-DWCNTs; and (f) sectional view of X-DWCNTs with high magnification. Arrowed rulers in b and e highlighted pores of about 70 μm in diameter (in yellow, star tags) and of about 40 μm in diameter (in cyan, hash tags), respectively, on pristine DWCNT and X-DWCNT scaffolds.

In the first place, the porous structure of the 3D materials was studied by SEM. SEM images of the top and cross-sectional views were acquired and developed a fully interconnected structure of irregularly shaped pores with a variable pore size distribution, ranging from tens to hundreds of micrometers in diameter (Figure 4). The cross-sectional views (Figure 4b, e) showed that the pores were interconnected by random paths within the scaffolds (pristine and cross-linked DWCNTs in the example), which has been demonstrated to be beneficial for neuronal growth and differentiation.^39,43,44,55^ Furthermore, high magnification images (Figure 4c, f) confirmed the presence of a network of randomly entangled DWCNTs arranged onto 3D pores’ facets. Control materials (Y-DWCNTs and Z-DWCNTs) developed porous structures with similar features (see Figure S4). From these results, we can conclude that cross-linking does not play a decisive role in the final pore size and structure of the 3D scaffolds (nor electrical conductivity) but, presumably, could affect their mechanical properties.

Because during the development of 3D conductive materials the electrical conductivity decreases as the porosity increases,^56,57^ studies on the changes in electrical conductivity during the development of 3D porous scaffolds were performed. During these studies, the main handicap was related to the measurement of the thickness of the 3D scaffolds. Due to the softness of the templated materials, achieving accurate results by using the High-Accuracy Digimatic micrometer was very difficult, and the thickness was deduced in this case from the cross-section views of the SEM images (see Figure S5). According to these data, the mean values determined for the electrical conductivity were, 22368 S/m, 8131 S/m, 5552 S/m, and 6596 S/m for pristine DWCNTs, X-DWCNTs, Y-DWCNTs, and Z-DWCNTs, respectively. It is important to remark that due to the heterogeneity and roughness of the 3D scaffolds that we obtain by using a simple glass microfiltration setup for their manufacturing, the emphasis of these studies was the correlation of the electrical properties and the microstructure of the 3D scaffolds, instead of emphasizing the absolute values of electrical conductivity. In these cases, we assume that the four-point probe may give inaccurate results due to the heterogeneous thickness of the samples and arbitrary probe placement;^58^ however, no significant degradation of the electrical conductivity was observed during the preparation of the porous materials.

During the growth of neuro/glial networks, the mechanical properties of the cellular microenvironment play a critical role in cell development and behavior, and thus the mechanical behavior of our manufactured 3D scaffolds was studied.

The mechanical behavior of pristine, TEG-functionalized (Y-DWCNTs, Z-DWCNTs), and cross-linked (X-DWCNT) scaffolds was analyzed under tensile and compressive loading. For low-density CNT ensembles, such as developed 3D DWCNT scaffolds, any little variation in density can significantly affect the mechanical performance, because usually, samples with higher density exhibit higher force to break and, therefore, higher strength if normalized simply by the cross-section area. To directly evaluate any effect of TEG-functionalization and cross-linking on the tensile properties of DWCNT scaffolds, we hereby compare the tensile strength and modulus expressed in specific terms of MPa/SG, where SG is the specific gravity of the sample.

**Figure 5:**
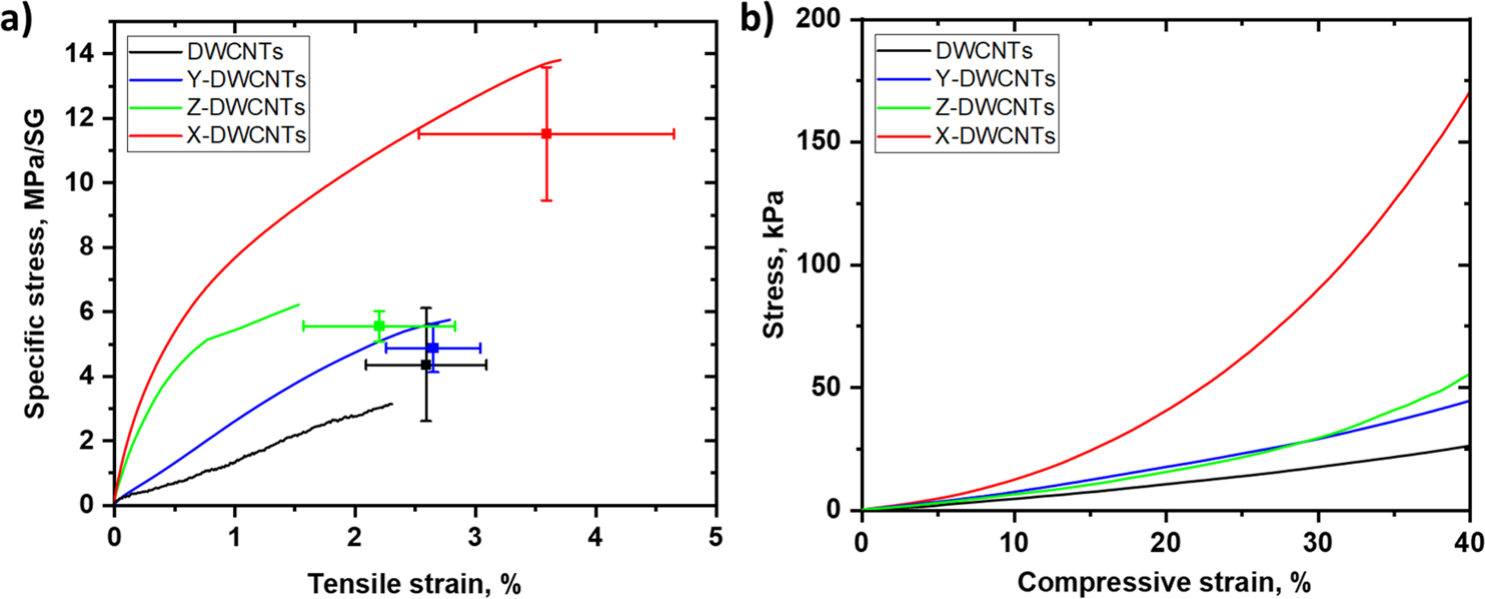
Representative (a) tensile (until rupture) and (b) compressive (up to 40% strain) stress–strain curves for the pristine (DWCNTs), TEG-functionalized (Y-DWCNTs, Z-DWCNTs), and cross-linked (X-DWCNTs) scaffolds.

The corresponding tensile stress–strain curves are presented in Figure 5a and mechanical characteristics are summarized in Table 1. All scaffolds demonstrate ductile behavior with visible yielding and plastic deformation region with average strain-to-break over 2%. Functionalization of the DWCNT scaffold with a single TEG chain (Y-DWCNTs) does not result in a notable change in tensile performance; however, the introduction of two chains (Z-DWCNTs) shifts the strength and modulus to the higher range (5.6 MPa/SG and 725 MPa/SG, respectively) compared with the pristine scaffolds (4.4 MPa/SG strength and 200 MPa/SG modulus). Cross-linking post treatment induces the most significant change in both the strength and stiffness of the scaffolds. Comparing the characteristics expressed in specific terms, we understand that the notable changes in the tensile performance of the X-DWCNTs scaffolds are not associated simply with a gain in density but with more efficient stress transfer between cross-linked CNTs within a 3D network. The typical stress–strain curve of the cross-linked scaffold exhibits mush steeper slope in comparison with the pristine one (DWCNTs) and TEG-functionalized scaffolds (Y-DWCNTs, Z-DWCNTs), with the specific modulus increase to 1.1 GPa/SG. The DWCNT scaffolds are stiffer than other polymeric scaffolds (with the modulus in the approximately kilopascal to a few megapascal range)^59^ but significantly softer than typical silicon- (∼200 GPa, silicon Utah array ∼150 GPa), tungsten- (∼200 GPa), iridium- (528 GPa),^41^ and other metal microwire-based implantable electrodes and neural probes, and therefore, with less pronounced mechanical mismatch with the spinal cord tissue (up to ∼1–2 MPa tensile modulus^60^). In addition, cross-linked DWCNT scaffolds exhibit a higher tensile strength in comparison with the pristine ones (11.5 MPa/SG versus 4.4 MPa/SG). Most importantly, they demonstrate ductile behavior manifested by the pronounced elongation to failure of 3.6% retained after cross-linking.

**Table 1:** Tensile and Compressive Properties of the Pristine (DWCNTs), TEG-Functionalized (Y-DWCNTs, Z-DWCNTs), and Cross-linked (X-DWCNTs) Scaffolds

		tensile properties			compressive properties at 40% strain	
scaffold	apparent volumetric density (g/cm^3^)	specific strength (MPa/SG)	specific modulus (MPa/SG)	strain-to-break (%)	strength (kPa)	modulus (kPa)
DWCNTs	0.13	4.4 ± 1.7	200 ± 55	2.6 ± 0.5	25	30
Y-DWCNTs	0.15	4.9 ± 0.7	249 ± 16	2.7 ± 0.4	44	65
Z-DWCNTs	0.10	5.6 ± 0.5	725 ± 431	2.2 ± 0.6	55	50
X-DWCNTs	0.34	11.5 ± 2.0	1100 ± 575	3.6 ± 1.1	170	87

The behavior of DWCNT scaffolds under compressive loading was assessed on the representative samples (one of each type). The obtained compressive stress–strain curves are presented in Figure 5b and Figure S6. In general, the scaffolds exhibit similar behavior to low-density porous CNT networks (MWCNTs scaffolds with vapor phase polymerized polypyrrole for neural prostheses,^39^ aligned CNT foams^61^) and other foam-like materials such as cellulose nanofibril aerogels.^62^ At low deformations (up to 3–4% strain), all samples show linear regime, which is elastic compression of CNT scaffold. It is followed by further limited augmentation of compressive stress and plastic yielding toward to higher strain range. Above the 40–80% strain range, the compressive stress increases abruptly, indicating the progressive irreversible deformation through densification and stiffening until the entire collapse of the highly porous structure of DWCNT scaffolds. The pristine and single TEG chain functionalized (Y-DWCNTs) scaffolds show high compressibility, with the ultimate compressive failure event at 96–98% strain, attesting to high porosity of the structure. In contrast, scaffolds functionalized with two TEG chains (Z-DWCNTs) and cross-linked (X-DWCNTs) demonstrate reduced strain to failure (65–70%) possibly due to stronger interactions between CNTs at points of contact. Comparing stress levels for identical strain range (up to 40%), the compression performance of scaffolds is improved dramatically after cross-linking reaction, reaching stress of 170 kPa. This stress level is almost 7 times higher than that of the pristine scaffold, whereas the gain in density by cross-linking is only 2.7 times (0.346 g/cm^3^ versus 0.128 g/cm^3^ for the pristine). X-DWCNTs scaffolds can tolerate significant mechanical compression through cross-linked CNT network, at the same time retaining elasticity. The modulus (minimum compressive modulus) determined at the initial compression stage of the scaffolds is in the range 30 to 90 kPa, and similar to other materials used in neuronal regeneration, such as cross-linked graphene-based polyacrylamide hydrogels (30–50 kPa),^63^ MWCNTs/polypyrrole scaffolds (50–200 kPa),^39^ porous PDMS polymeric scaffolds (45 kPa),^44^ graphene scaffolds for neural stem cells growth in treating spinal cord injuries (30–64 kPa),^40^ and with potential suitability for spinal cord restoration (elastic modulus 40 kPa, maximum compressive failure stress 62 kPa).^64^ The manufactured DWCNT scaffolds provides a balance between their mechanical (tensile and compressive) and electrical properties.

### 3.4 X-DWCNT Scaffold Favors the Formation of Microclusters of Primary Hippocampal Neurons

**Figure 6:**
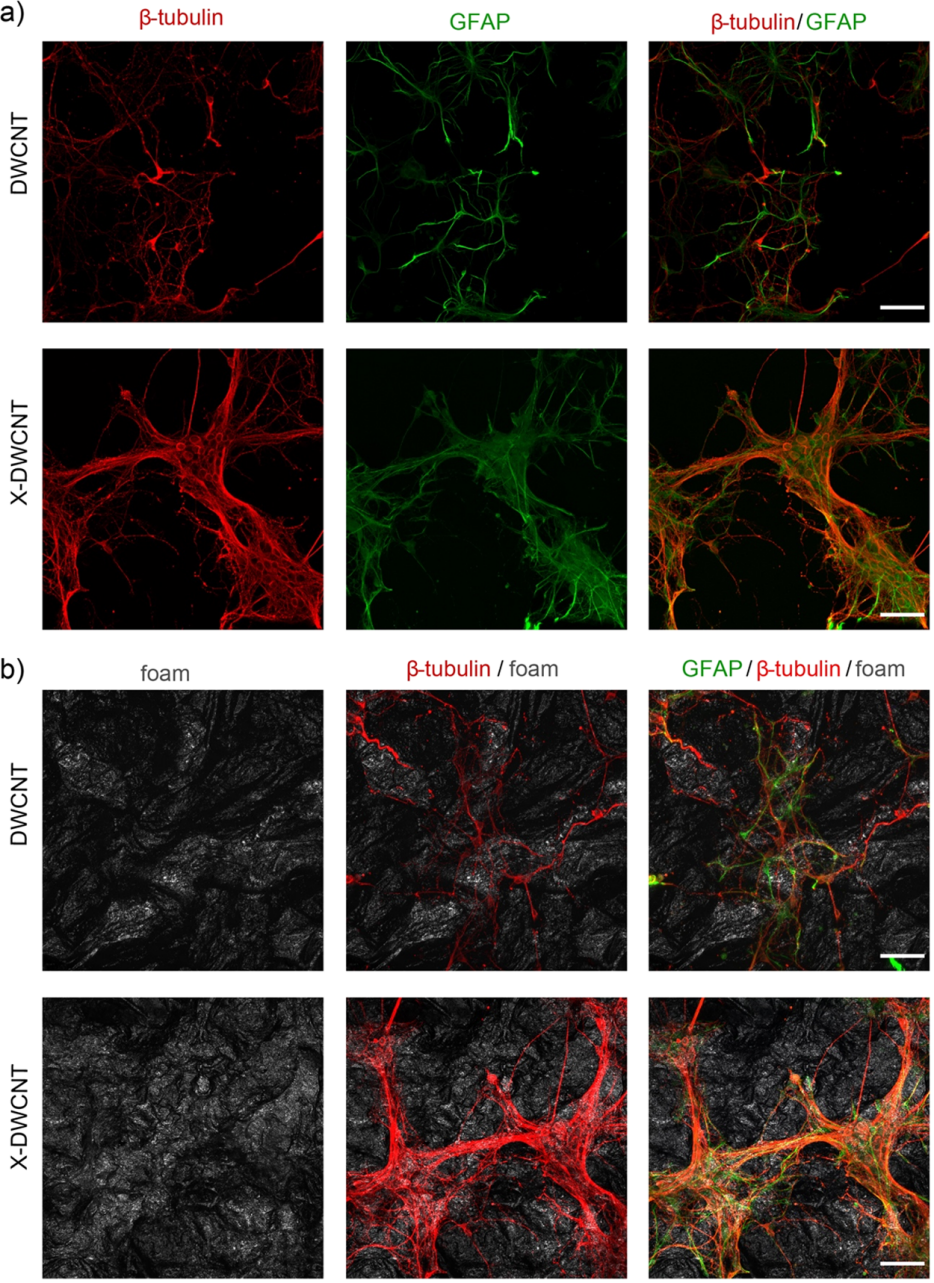
Primary hippocampal cultures developed on DWCNT and X-DWCNT scaffolds. (a) Confocal micrographs show hippocampal cultures grown (8 DIV) on DWCNTs (top) and X-DWCNTs (bottom) immune-stained for β-tubulin III (red) and GFAP (green), scale bar 50 μm. (b) Confocal reconstructions of DWCNTs and X-DWCNTs structure (in gray the two materials highlighted collecting the reflected light); neurons (red) and glial cells (green) colonized the curved structure of the foams differently, clustering more on X-DWCNTs. Scale bar 50 μm.

We tested pristine DWCNTs and X-DWCNTs 3D scaffolds by culturing hippocampal cells for 8–10 days. In both culture groups, we visualized neurons and neuronal network morphology by immunofluorescence microscopy of β-tubulin III, a neuronal cytoskeletal marker.^14,44^ Similarly, glial cells were identified by glial fibrillary acidic protein (GFAP), an astrocyte cytoskeletal marker.^44^Figure 6a shows the presence of neurons (in red) and GFAP-positive astrocytes (in green) on both substrates and it is supportive of DWCNT and X-DWCNT biocompatibility, both scaffolds allowing cell adhesion and survival. Two major differences in the topography of hippocampal cultures emerged when comparing the two substrates, highlighted in the Figure 6 confocal micrographs., First, neurons adherent to DWCNT developed traditional networks comprising sparse cells connected by long axonal processes; second, in DWCNT, neurons displayed only partial superimposition with glial cells, characterized by stellate morphology (Figure 6a, top panels). In X-DWCNT neurons were organized in small and tightly interconnected clusters, mostly adherent on carpets of large and flat glial cells (Figure 6a, bottom panels). The ability of X-DWCNT-interfaced cells to follow the curved structure of the porous apparently allowed a higher biocolonization of the material, shown in Figure 6b, where the foams were made visible by confocal microscopy collecting the reflected light (in gray).

### 3.5 Live Calcium Imaging of Hippocampal Neurons Developed on DWCNT and X-DWCNT

**Figure 7:**
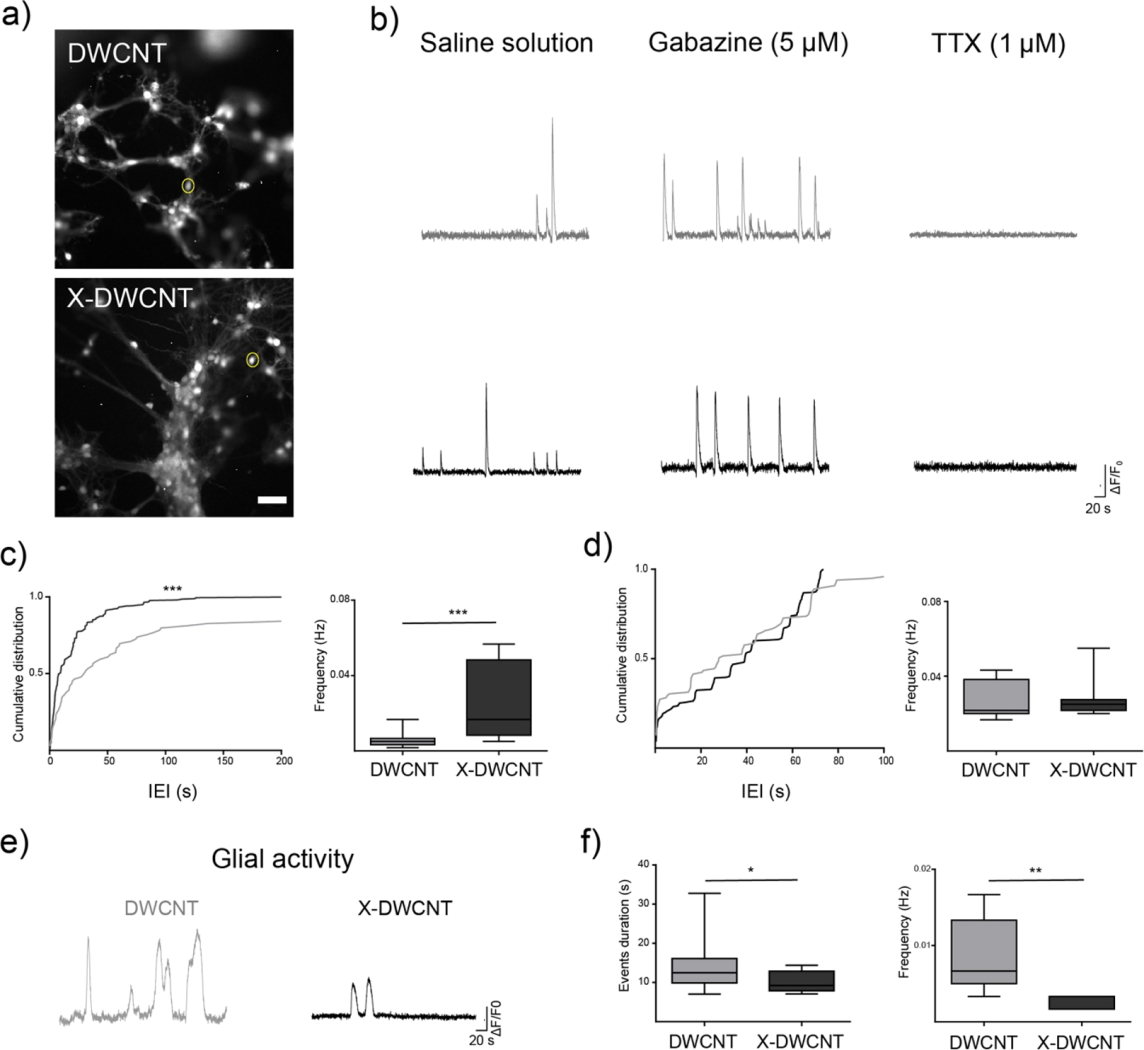
Live calcium imaging of neuron and glial cells developed on 3D DWCNT. (a) Snapshots of representative fields of hippocampal cultures after 8–10 DIV on DWCNT (top) and X-DWCNT (bottom); single cells are stained by the calcium dye Oregon Green 488-BAPTA-1 AM. Dashed lines indicate examples of selected regions of interest (ROI). Scale bar: 50 μm. (b) Spontaneous sample traces of Ca^2+^ events recorded on DWCNT (light gray, top) and X-DWCNT (in black, at the bottom), respectively, in saline solution (left traces), gabazine (central traces), and tetrodotoxin (right traces). TTX administration confirms the neuronal nature of cells sampled in these recordings. (c) Cumulative probability plot of interevents interval (IEI, left), and the box plot of event frequency, right. (d) Cumulative probability plot of IEI in gabazine (left), and the box plot of event frequency (right). (e) Representative traces of glia calcium events on DWCNT (in light gray, left) and on X-DWCNT (in black, right). (f) Glial cell activities detected in TTX and compared between the two scaffolds in terms of event duration (left) and frequency (right).

To investigate how the interplay between DWCNT and X-DWCNT scaffolds and the topography of neuronal circuits affected neuronal signaling, we live-monitored spontaneous synaptic activity, emerging from small groups of neurons, by fluorescence calcium imaging (see Movies S1 and S2).^44^ Consistent with data from confocal images, fluorescent neurons visualized by loading them with the calcium dye Oregon Green 488- BAPTA-1 AM, when cultured on X-DWCNT, appeared to colonize differently the scaffold when compared to those grown on DWCNT (Figure 7a). The size of the imaged sampled fields (409.6 μm × 409.6 μm) enabled simultaneous monitoring and subsequent analysis of comparable amounts of neurons in each field (8 ± 2 cells in DWCNT and 12 ± 3 in X-DWCNT; three different culture series). Figure 7b reports representative fluorescent tracings of spontaneous calcium activity recorded in DWCNT (top, light gray, see Movie S1) and in X-DWCNT (bottom, black; see also Movie S2). As previously reported,^44^ in hippocampal neurons, spontaneous synaptic activity appears as the occurrence of transient episodes of cytoplasmic calcium elevation. We measured the occurrence of spontaneous calcium events in neurons by calculating their inter events interval (IEI). Neurons cultured on X-DWCNT display IEI of 19.5 ± 1 s, a value significantly lower when compared to control neurons cultured on DWCNT (102.2 ± 19.8 s), shown in Figure 7c, left, as cumulative distributions (*n* = 24 cells for DWCNT and *n* = 39 cells for X-DWCNT; Kolmogorov–Smirnov test, *p* < 0.001). The lower IEI in X-DWCNT was in fact accompanied by higher calcium events frequency in X-DWCNT compared to DWCNT (box plot in Figure 7c, right; on average 0.006 ± 0.008 Hz in DWCNT, *n* = 24 cells and 0.027 ± 0.003 Hz for X-DWCNT, *n* = 39 cells, no parametric data, Mann–Whitney test, *p* < 0.001). These data suggest that the X-DWCNTs, by favoring the formation of small clusters of neurons, thus increasing the colonization of cells along the curves of scaffold surfaces, enable higher network outputs. To gain insights into the dynamics of the two different DWCNT-network constructs, we pharmacologically blocked synaptic inhibition by application of the GABA_A_ receptors antagonist gabazine (5 μM), a manipulation known to unmask stereotyped network activity patterns.^14,44^ In both DWCNT and X-DWCNT groups, with respect to the spontaneous activity, the removal of synaptic inhibition induced the emergence of regular calcium oscillations with a similar pace in the two conditions (Figure 7b, central traces). Traces analysis revealed similar IEIs (35 ± 3 s for DWCNT and 37 ± 1 s for X-DWCNT, *p* = 0.06) and frequencies (0.025 ± 0.003 Hz for DWCNT and 0.026 ± 0.001 Hz for X-DWCNT, no parametric data, Mann–Whitney test, p = 0.15), as shown in Figure 7d as cumulative distribution and box plots, respectively. This result supports the idea that diverse networks developed when interfaced to the two materials. Specifically, while on DWCNT networks were composed by sparse neurons, on X-DWCNT neurons were organized in small clusters of cells, and their activity was differently shaped by synaptic inhibition. Interestingly, when applying tetrodotoxin (TTX 1 μM, a known blocker of action potential mediated-synaptic activity;^44^) we completely removed neuronal activity (Figure 7b, right traces) disclosing glial cells spontaneous calcium signaling (Figure 7e)^65^ recorded as slow calcium episodes of longer duration in DWCNT samples when compared to X-DWCNT ones (13.5 ± 5 s for DWCNT and 10.1 ± 2.6 s for X-DWCNT, no parametric data, Mann–Whitney test, *p* < 0.05), as summarized by the box plot in Figure 7f, left. Interestingly, the frequency of glial events in pristine DWCNT was higher when compared to X-DWCNT one (0.008 ± 0.001 Hz for DWCNT, *n* = 8 cells, and 0.002 ± 0.0003 Hz for X-DWCNT, *n* = 7 cells; Figure 7f, right, *p* < 0.01).

**Figure 8:**
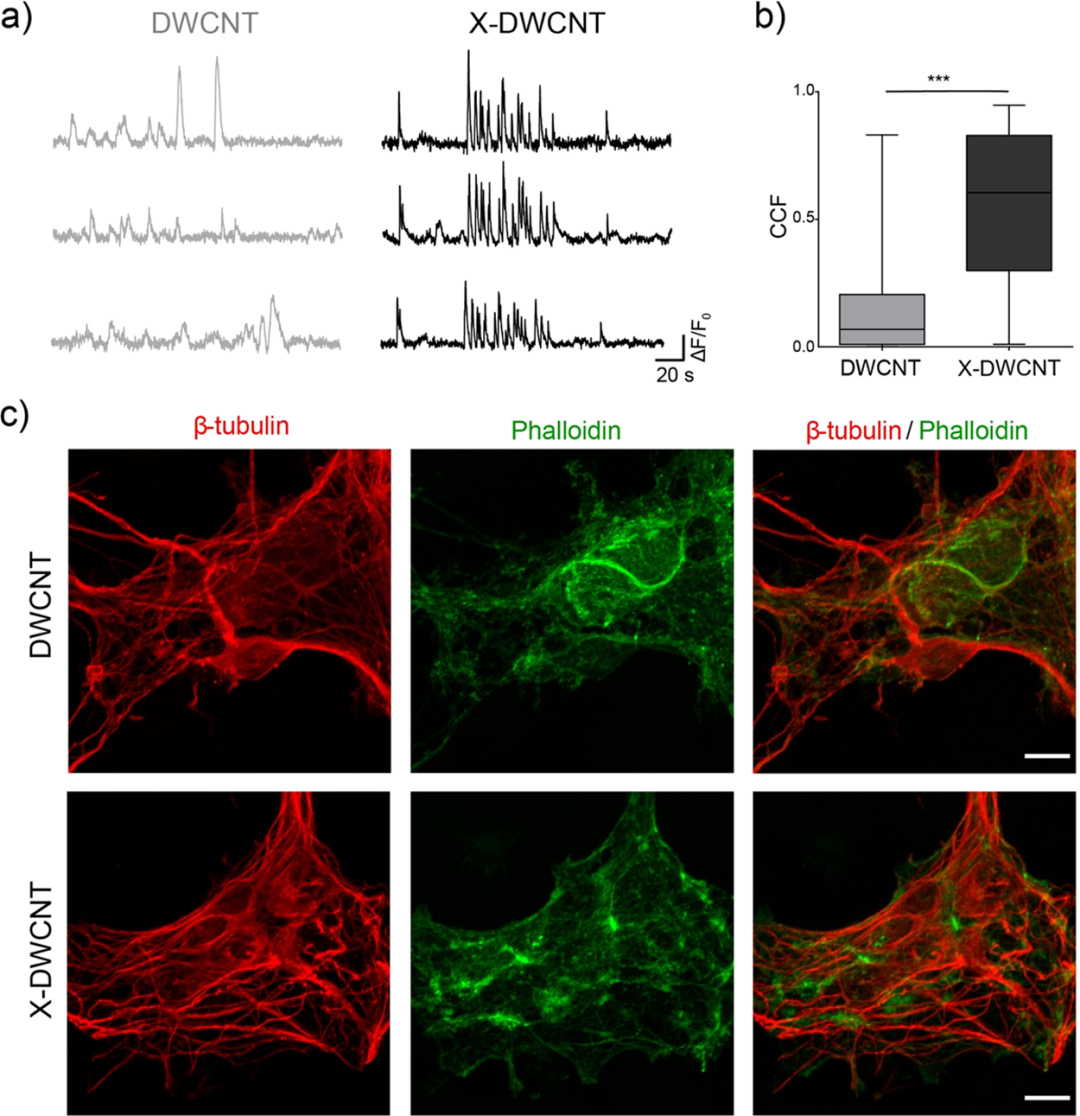
Neuronal activity cross-correlation in networks supported by 3D DWCNT scaffolds. (a) Spontaneous calcium events recorded from three different cells grown interfaced to DWCNT (light gray, left) and X-DWCNT (black, right). (b) Bar plot summarizing the cross-correlation factor (CCF) between the two data sets. (c) Confocal reconstructions of DWCNT- and X-DWCNT-interfaced neurons visualized by β-tubulin III (in red) with actin filaments highlighted by phalloidin staining (in green). Scale bar 10 μm.

In a different set of experiments, we further investigated in DWCNT and X-DWCNT the emergence of synchronization of spontaneous calcium episodes among different neurons located within the same visualized field evaluating the cross-correlation function (CCF). Figure 8a shows sampled tracings taken from distant cells within the same recording field. Pooling together the data revealed that cells are significantly more correlated on X-DWCNT (CCF 0.6 ± 0.01) when compared to DWCNT ones (CCF 0.1 ± 0.01), as summarized in the bar plots in Figure 8b (no parametric data, Mann–Whitney test, *p* < 0.001). Thus, X-DWCNT affects the neuronal and glial network topography and activity. Finally, to verify whether the differences in the calcium events were due to a different organization of the cytoskeleton actin filaments, critically involved in sensing physical cues, we performed immunofluorescence for phalloidin, a marker of polymerized actin. As shown by the representative confocal micrographs in Figure 8c, no apparent changes emerged in this respect.

### 3.6 Characterization of Neuronal Cultures Interfaced to Y-DWCNT and Z-DWCNT Scaffolds

To evaluate the impact that DWCNT cross-linking could have on neuronal activity, we tested the ability of unlinked Y-DWCNT and Z-DWCNT control 3D scaffolds to support the development of a mature and functional neuronal network. Interestingly, we discovered that in the absence of cross-linking, neither scaffold provided support for cell development, survival, and network formation for more than a few days. That prevents us from performing any functional evaluation at DIV 8–9, the time point at which pristine and X-DWCNTs scaffolds have been tested in their neuronal network organization and neuronal activity. Specifically, we observed a fast-rising cell degradation that reduced cell density, viability, and network dimension when hippocampal cells were interfaced to such materials for more than 4 DIV, precluding later formation of synaptic activity (see Figure S7b–d for Y-DWCNT and Figure S7f–h for Z-DWCNT). The limited cell survival and the absence of neuronal network maturation seems presumably related to the detrimental effect on cell adhesion and maturation of the very high number of immobilized chemical groups inserted in the carbon nanotube external wall (see FGC for Y-DWCNT and Z-DWCNT in section 3.2). This condition is attenuated in the X-DWCNT cross-linked scaffold by the involvement of the reactive chemical groups in establishing carbon nanotube bridging.

## 4 Conclusions

In this work, highly conductive DWCNTs are employed for the development of 3D neural scaffolds that show promising properties for their application in future smart interfaces. Chemical cross-linking approaches with TEG derivatives have allowed for improvement of the scaffold’s mechanical properties, both under tensile and compressive loads, while a simple NaCl crystal templating approach has provided 3D self-standing scaffolds with interconnected porosity. Immunofluorescence studies have demonstrated that cell adhesion and survival are possible in both DWCNT and X-DWCNT scaffolds, with the latter allowing a higher biocolonization of the material and the formation of a connected network following the scaffold 3D morphology. Indeed, the 3D organization of X-DWCNT interfaced circuits is also associated with a higher degree of synchronized synaptic activity.

The alteration in the neuronal activity of this system could not be related to a unique factor but, instead, to the synergic contribution of many features. Indeed, the differences observed between pristine DWCNT and X-DWCNT samples could be associated with (i) the different network organization (i.e., cell clustering); (ii) the altered electrical structure of the outer DWCNT wall induced by the functionalization; (iii) the chemical contribution of the cross-linking molecular bridges; (iv) the altered mechanical properties of the 3D scaffolds. However, in our opinion, the most evident change we observed between the two conditions is the 3D topological organization of the network induced by X-DWCNT substrates. Consequently, we are prone here to consider this aspect as the key element responsible for the higher network output. Specifically, we favor the hypothesis that the cross-linked material promotes a better three-dimensional organization of the neuronal network, allowing the formation of neuronal clusters, which translates into boosted network activity.^44^

Overall, these findings demonstrate how chemical tailoring can be used to tune the properties of CNT-based scaffolds while simultaneously controlling the neuronal and glial network topography and activity and is a promising avenue for future research in conductive components of neural interfaces.

## Supporting Information Available

Scheme S1. Synthetic approach for the control materials. Scheme S2. Synthetic approach for amino-TEG 2 and amino-TEG 3. Figure S1. Raman maps are provided for pristine DWCNTs and cross-linked DWCNTs (X-DWCNTs). Figure S2. Raman spectra for the control materials. Figure S3. TGA curves for the control materials. Figure S4. SEM images for the control materials. Figure S5. SEM analysis and measurement of the cross sections for the 3D scaffolds. Figure S6. Compressive stress–strain curves recorded until the entire collapse of scaffolds’s porous structure. Figure S7. Neuronal studies for the control materials. Figure S8. Neuronal development at different planes. Figure S9. Photos of the DWCNTs specimens tested in tension and compression. Figure S10. Example of change of displacement recorded at compressive test (DOC)
Movie S1, sequence of Ca^2+^ images highlighting calcium activity in pristine DWCNT (MP4)
Movie S2, sequence of Ca^2+^ images highlighting calcium activity in cross-linked X-DWCNT (MP4)
