# Supplementary material for: TEGylated Double-Walled Carbon Nanotubes as Platforms to Engineer Neuronal Networks
Source: ACS Appl Mater Interfaces. 2022 Oct 21;15(1):77–90. doi: 10.1021/acsami.2c16808 (PMC9837783; doi:10.1021/acsami.2c16808)
Supplement: Supplementary file 1 — am2c16808_si_001.doc [file am2c16808_si_001.doc]

Supporting information

TEGylated Double-walled Carbon Nanotubes as Platforms to Engineer Neuronal Networks

Myriam Barrejón,^a,b*^ Francesca Zummo,^c^ Anastasiia Mikhalchan,^d^ Juan J. Vilatela,^d^ Mario Fontanini,^c^ Denis Scaini,^c,e,f^ Laura Ballerini,^c*^ and Maurizio Prato ^a,e,g*^

^a^ Department of Chemical and Pharmaceutical Sciences, INSTM, UdR Trieste, University of Trieste, Via L. Giorgieri 1, 34127 Trieste, Italy

^b^ Neural Repair and Biomaterials Laboratory, Hospital Nacional de Parapléjicos (SESCAM), Finca la Peraleda s/n, 45071 Toledo, Spain.

^c^ International School for Advanced Studies (SISSA/ISAS), Trieste 34136, Italy

^d^ IMDEA Materials, Eric Kandel 2, 28906 Getafe, Madrid, Spain.

^e^ Basque Foundation for Science, Ikerbasque, Bilbao 48013, Spain

^f^ University of Basque Country, Faculty of Pharmacy, Paseo de la Universidad 7, 01006 Vitoria-Gasteiz, Spain

^g^ Center for Cooperative Research in Biomaterials (CIC biomaGUNE), Basque Research and Technology Alliance (BRTA), Paseo de Miramon 194, 20014 Donostia San Sebastián, Spain


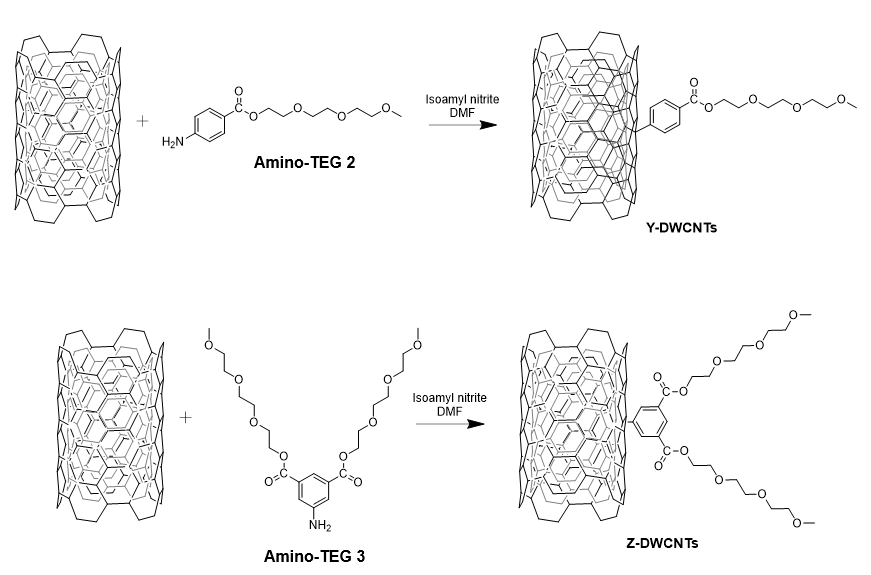


**Scheme S1.** Synthetic strategy for the preparation of TEG-functionalized DWCNTs (Y-DWCNTs and Z-DWCNTs).


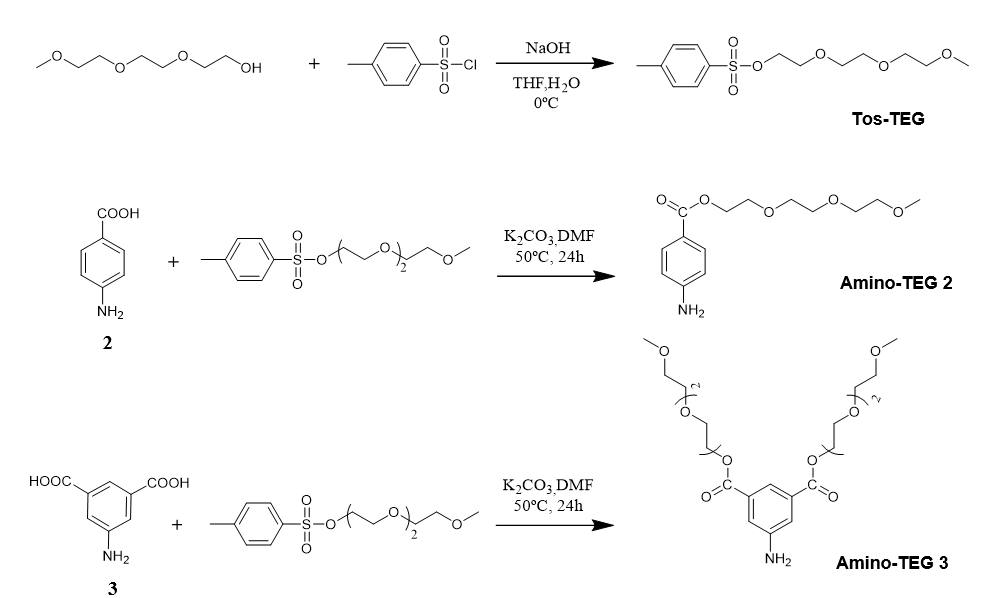
**Scheme S2.** Synthetic strategies for the preparation of Tos-TEG, Amino-TEG **2** and Amino-TEG **3**.


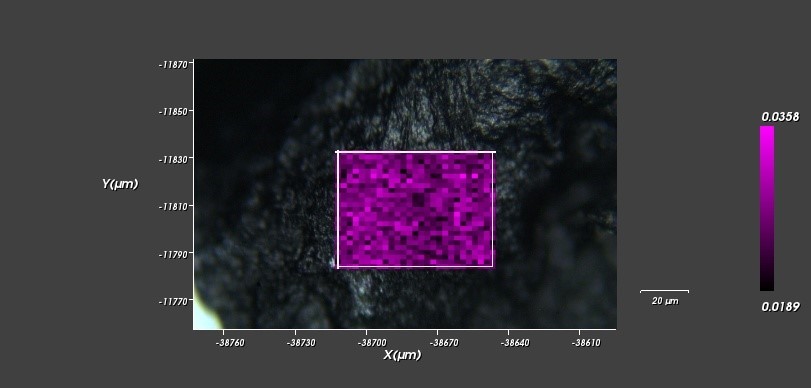


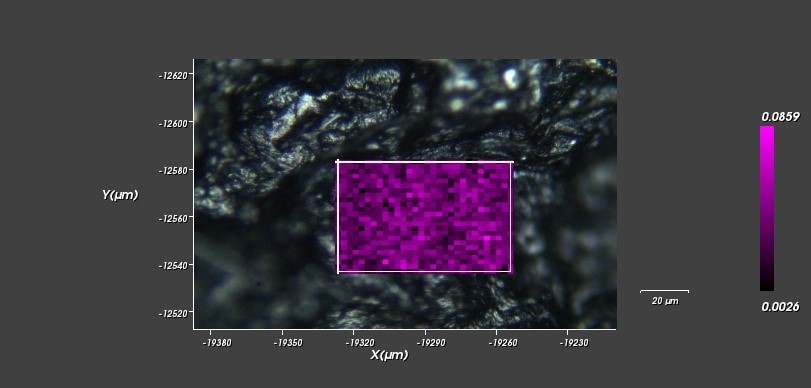


**Figure S1.** Raman maps of *I_D_/I_G_* ratio acquired with 785 nm laser for pristine DWCNTs (top) and X-DWCNTs (bottom).


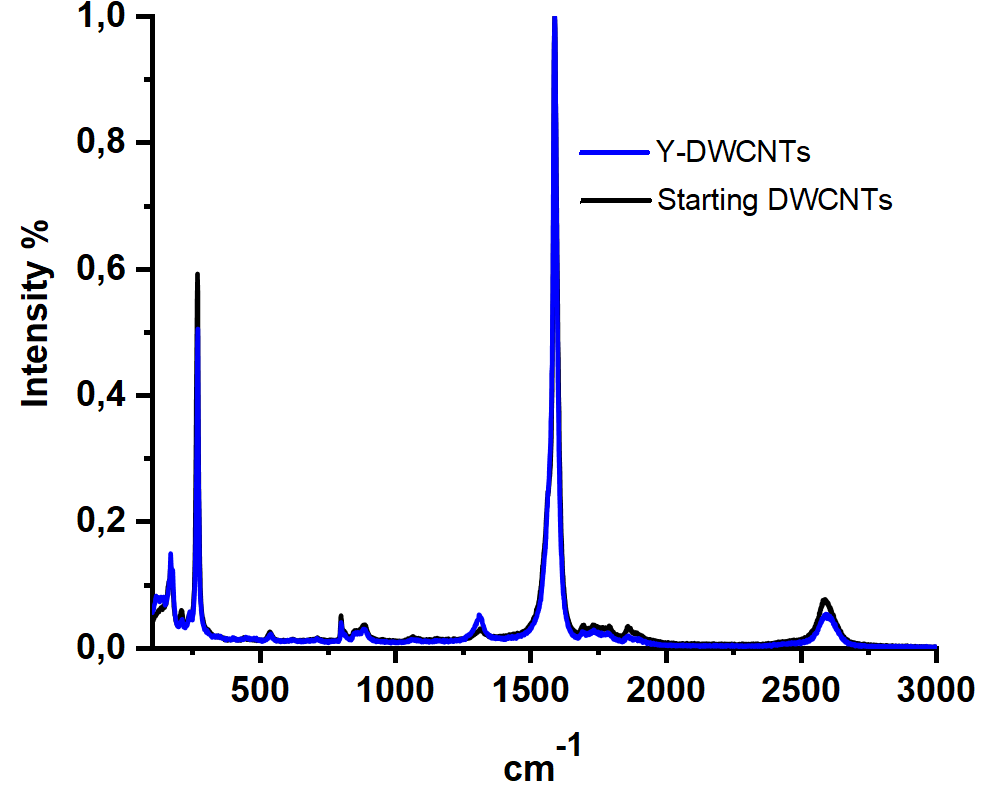

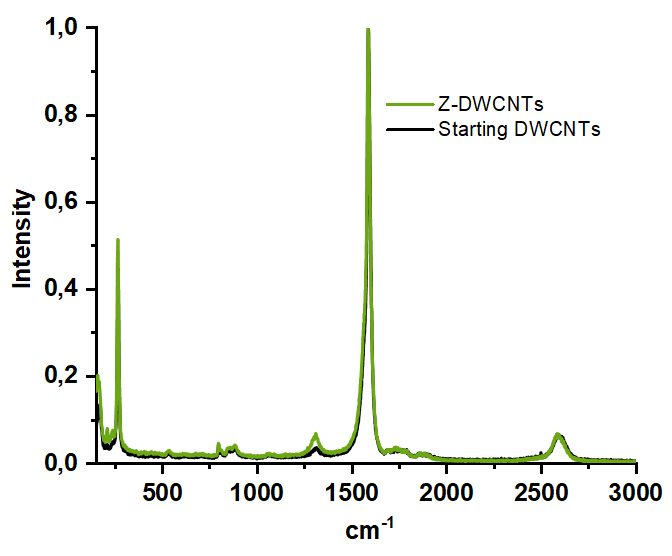


**Figure S2.** Extended Raman spectra for the control materials Y-DWCNTs (left) and Z-DWCNTs (right) compared to the starting DWCNTs.


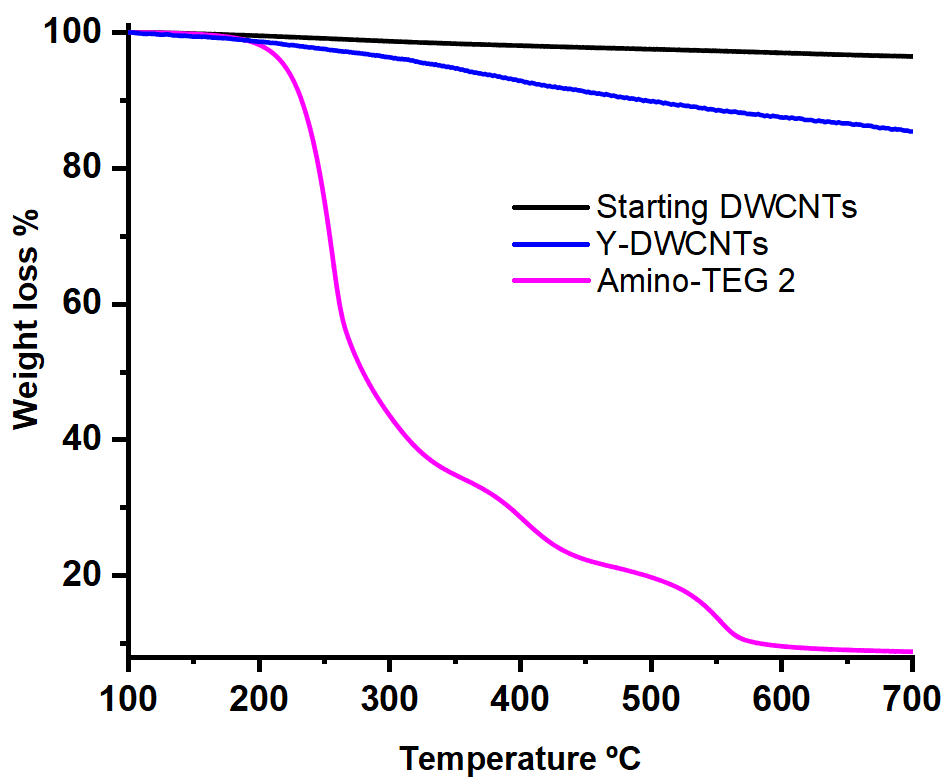

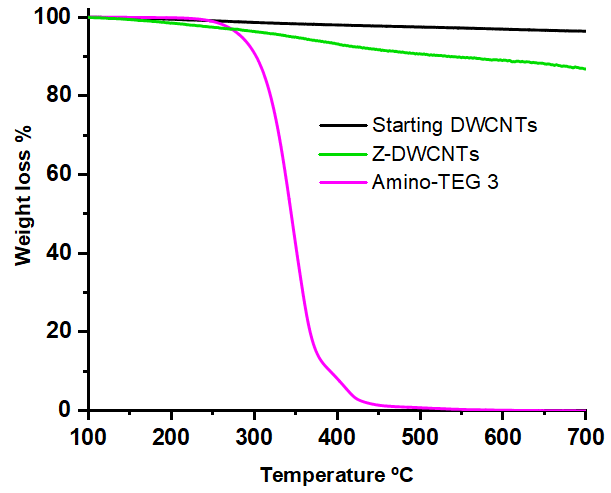


**Figure S3.** TGA curves for Y-DWCNTs (left) and Z-DWCNTs (right) compared to the starting DWCNTs and the corresponding amino-TEG derivatives.


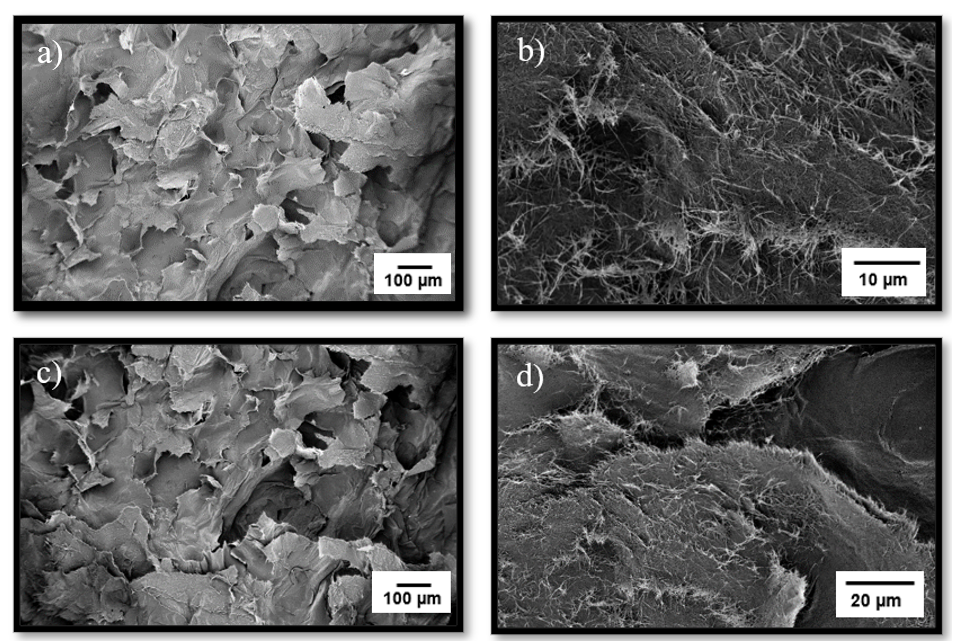


**Figure S4.** SEM images for the control materials: a) and b) sectional view and high magnification for Y-DWCNTs; c) and d) sectional view and high magnification for Z-DWCNTs.

**Figure S5.** SEM analysis and measurement of the cross-sections for a) pristine DWCNTs, b) Y-DWCNTs, c) Z-DWCNTs, and d) X-DWCNTs (samples fixed by carbon
sticky tape to the metallic holder).


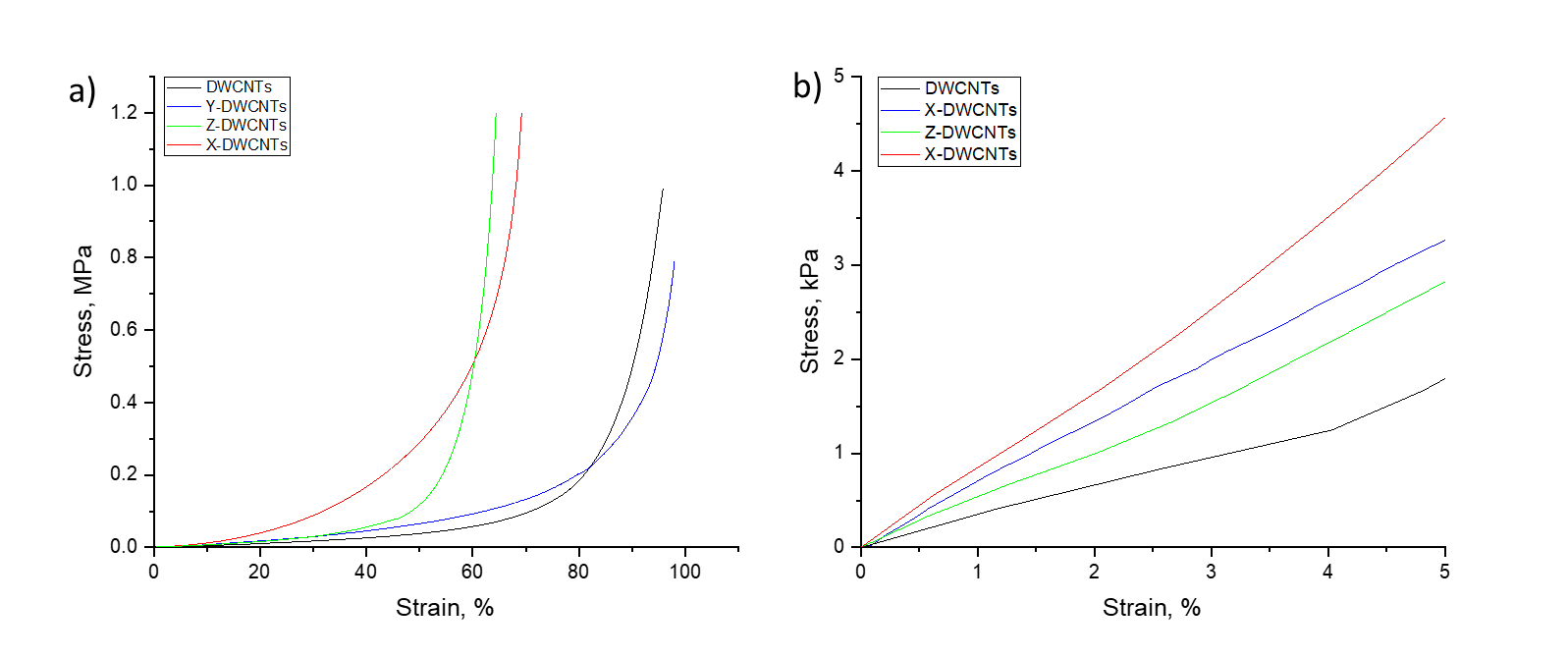


**Figure S6.** a) Compressive stress-strain curves recorded till permanent collapse of the scaffolds’ porous structure; b) enlarged initial linear region for which the minimum compressive modulus was determined.


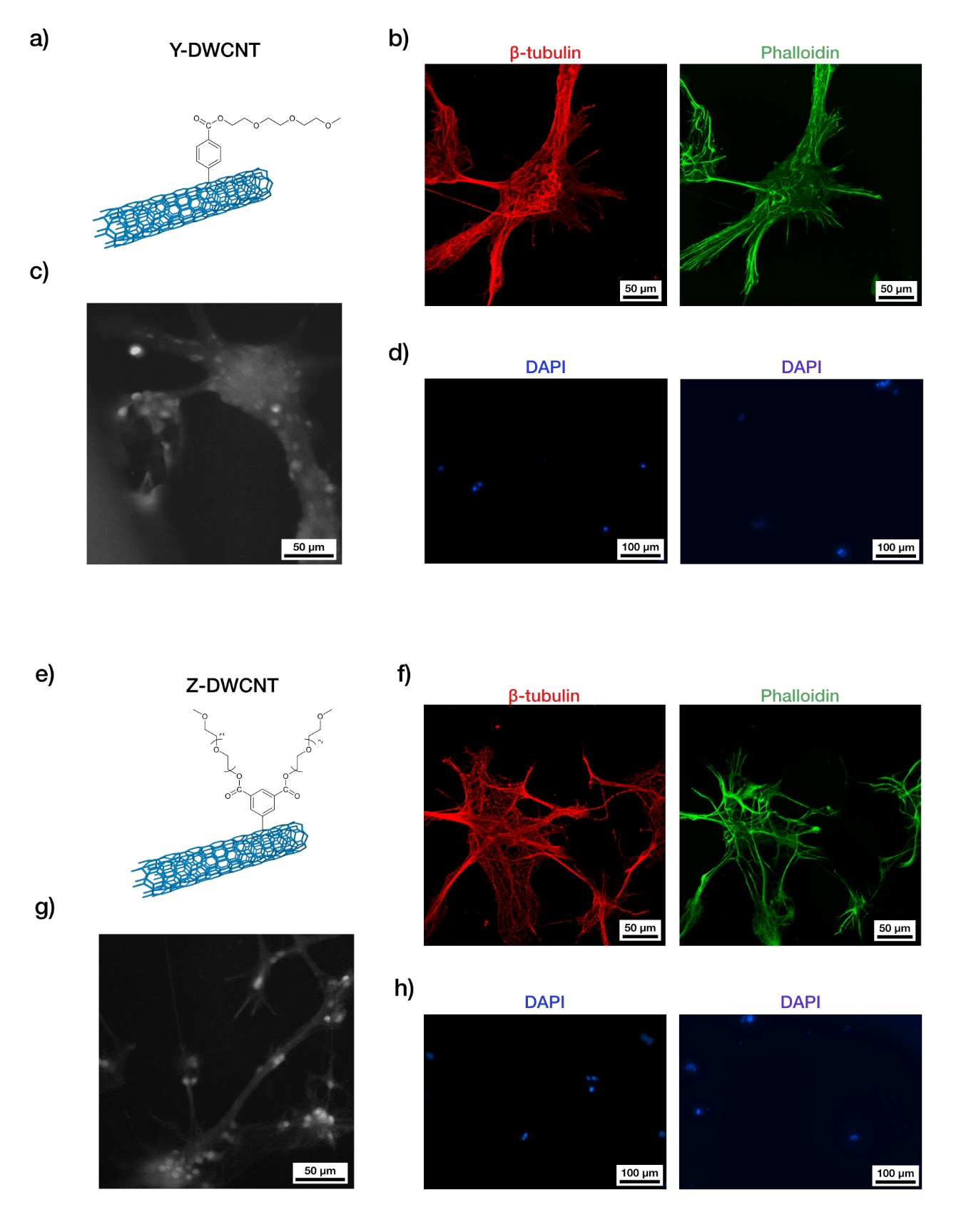


**Figure S7.** Neuronal cultures on no functionalized DWCNT scaffolds. a) sketch of the control material Y-DWCNT. In b), confocal micrographs showing hippocampal cultures grown (4 DIV) on Y-DWCNTs immune-stained for β-tubulin III (left, in red) and GFAP (right, in green). In c), a Ca^2+^ imaging representative frame of hippocampal cultures after 4 DIV on Y-DWCNT, cells are stained by the calcium dye Oregon Green 488-BAPTA-1 AM and, no calcium activity was present. In d), two examples of DAPI staining of the cell cultures on Y-DWCNT after 8 DIV. In e) the sketch of the control material Z-DWCNT. In f), confocal micrographs showing hippocampal cultures grown (4 DIV) on Z-DWCNTs immune-stained for β-tubulin III (left, in red) and GFAP (right, in green). In g), a Ca^2+^ imaging representative frame of hippocampal cultures after 4 DIV on Z-DWCNT, no calcium activity was present. In h), two examples of DAPI staining of the cell cultures on Z-DWCNT after 8 DIV.


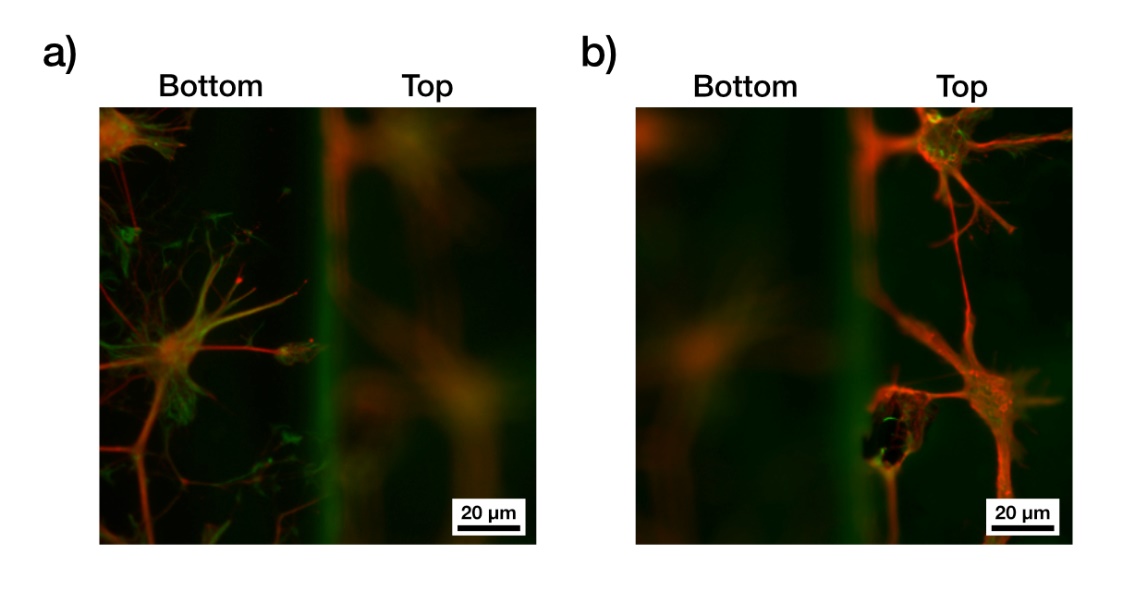


**Figure S8.** Neuronal cells localized at different planes above the scaffold. In a), an epifluorescence image showing hippocampal cultures let to develop for 8 DIV on a pristine DWCNTs scaffold immunostained for β-tubulin III (in red) and GFAP (in green). The in-focus part of the image (left) was situated on a lower level than the out of focus right part (the bottom of a pit on the scaffold’s surface). In b), the same field of view is shown refocusing on the right higher region. Despite the difficulty in acquiring optical images of the inner parts of the scaffold, a macroscopic 3D organization of the network is clearly visible.


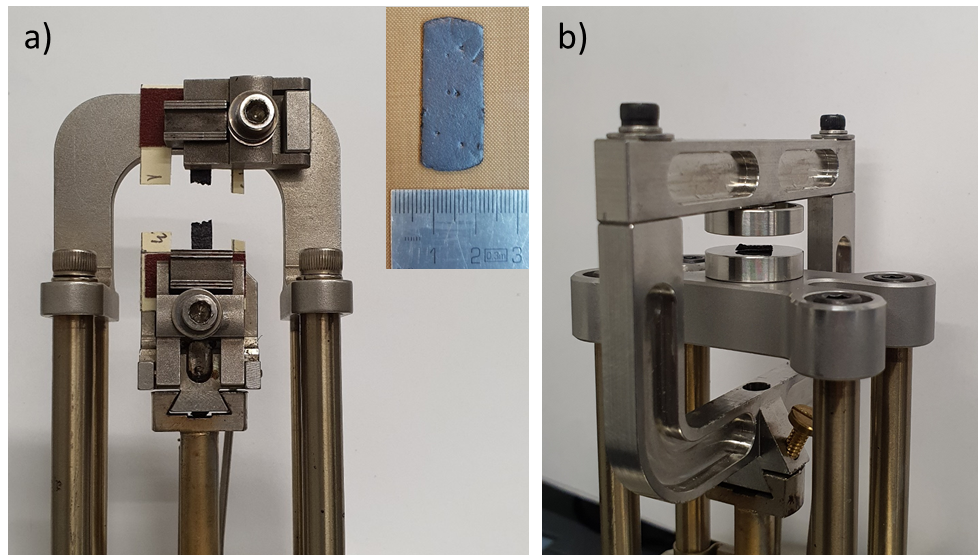


**Figure S9.** a) Example of the DWCNTs scaffold specimen fixed in the cardboard frame, photo is taken after tensile rupture. Inset: example of the typical manufactured freestanding and robust DWCNTs scaffold which can be easily handled and cut into rectangular specimens without premature structural damage. b) Example of the DWCNTs specimen placed in between the loading plates of the machine, photo is taken prior to compression test.


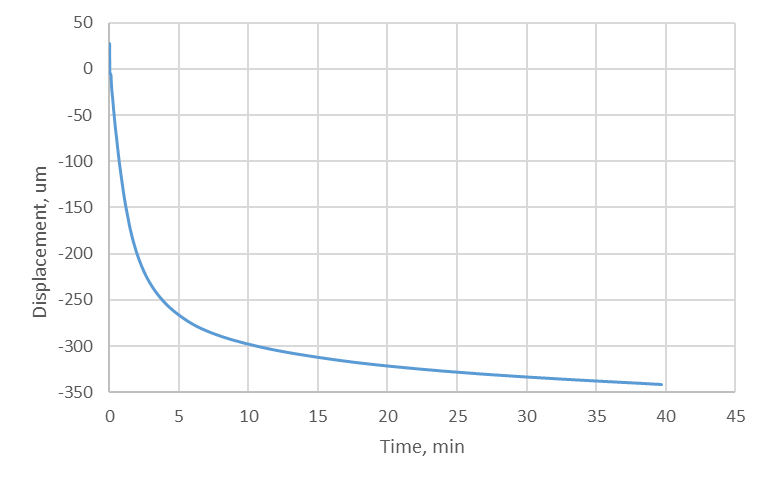


**Figure S10.** Example of the recorded change of displacement versus time indicating the compressive failure and entire collapse of the porous sample, at which the test was stopped.

**
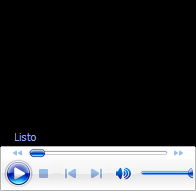
**

**Movie SM1.** A representative sequence of Ca^2+^ images highlighting calcium activity in pristine DWCNT interfaced cells (see Methods for details). Out-of-focus signals are due to cells placed at lower positions, presumably infiltrated within the 3D porous scaffold.


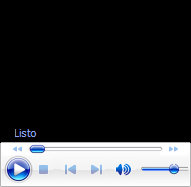


**Movie SM2.** A representative sequence of Ca^2+^ images highlighting calcium activity in cross-linked X-DWCNT interfaced cells (see Methods for details). Out-of-focus signals are due to cells placed at lower positions, presumably infiltrated within the 3D porous scaffold.
